# The reaction of organic peroxy radicals with unsaturated compounds controlled by a non-epoxide pathway under atmospheric conditions†

**DOI:** 10.1039/d2cp05166d

**Published:** 2023-02-23

**Authors:** Barbara Nozière, Olivier Durif, Eloé Dubus, Stephanie Kylington, Åsa Emmer, Fabienne Fache, Felix Piel, Armin Wisthaler

**Affiliations:** a KTH, Royal Institute of Technology, Department of Chemistry 114 28 Stockholm Sweden noziere@kth.se; b Université Lyon 1 and CNRS, UMR 5246, ICBMS 69626 Villeurbanne France; c University of Oslo, Department of Chemistry 0315 Oslo Norway

## Abstract

Today, the reactions of gas-phase organic peroxy radicals (RO_2_) with unsaturated Volatile Organic Compounds (VOC) are expected to be negligible at room temperature and ignored in atmospheric chemistry. This assumption is based on combustion studies (*T* ≥ 360 K), which were the only experimental data available for these reactions until recently. These studies also reported epoxide formation as the only reaction channel. In this work, the products of the reactions of 1-pentylperoxy (C_5_H_11_O_2_) and methylperoxy (CH_3_O_2_) with 2,3-dimethyl-2-butene (“2,3DM2B”) and isoprene were investigated at *T* = 300 ± 5 K with Proton Transfer Reaction Time-of-Flight Mass Spectrometry (PTR-ToF-MS) and Gas Chromatography/Electron Impact Mass Spectrometry. Unlike what was expected, the experiments showed no measurable formation of epoxide. However, RO_2_ + alkene was found to produce compounds retaining the alkene structure, such as 3-hydroxy-3-methyl-2-butanone (C_5_H_10_O_2_) with 2,3DM2B and 2-hydroxy-2-methyl-3-butenal (C_5_H_8_O_2_) and methyl vinyl ketone with isoprene, suggesting that these reactions proceed through another reaction pathway under atmospheric conditions. We propose that, instead of forming an epoxide, the alkyl radical produced by the addtion of RO_2_ onto the alkene reacts with oxygen, producing a peroxy radical. The corresponding mechanisms are consistent with the products observed in the experiments. This alternative pathway implies that, under atmospheric conditions, RO_2_ + alkene reactions are kinetically limited by the initial addition step and not by the epoxide formation proposed until now for combustion systems. Extrapolating the combustion data to room temperature thus underestimates the rate coefficients, which is consistent with those recently reported for these reactions at room temperature. While slow for many classes of RO_2_, these reactions could be non-negligible at room temperature for some functionalized RO_2_. They might thus need to be considered in laboratory studies using large alkene concentrations and in biogenically-dominated regions of the atmosphere.

## Abstract 2

Reactions of organic peroxy radicals (RO_2_) with alkenes at room temperature were found to proceed by the formation of large peroxy radicals (ROO-R’O_2_) instead of epoxides, which was reported until now for these reactions in combustion systems.

## Graphical abstract

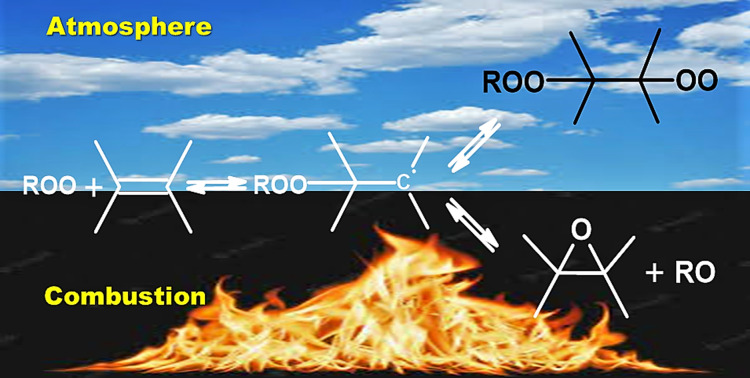

## Introduction

The cycles involving OH, HO_2_ and organic RO_2_ radicals in Earth's atmosphere are driving the atmosphere's oxidizing capacity. Over the last decades, trying to reconcile the atmospheric concentrations for these radicals with model predictions has led to important progress in the understanding of their chemistry. Numerous observations, under low-NO_*x*_ conditions and/or in vegetation-impacted regions,^1–6^ have reported elevated OH concentrations compared to model predictions, which was attributed to unidentified OH sources and RO_2_ sinks.^5,6^ These observations prompted numerous experimental and theoretical studies, which, in turn, led to the identification of many monomolecular reactions for the RO_2_ (H-shifts, cyclisation, *etc.*) regenerating OH without consuming NO.^7–14^ These reactions are now included in atmospheric chemical models. But, while they have reduced the discrepancies with atmospheric observations,^15^ some differences remain and unknown sinks for RO_2_ are still reported in the atmosphere.^5,6^ Discrepancies with models are also reported for laboratory studies, in particular at low NO_*x*_,^14^ which can also potentially be due to unidentified RO_2_ reactions.

**Scheme 1:**
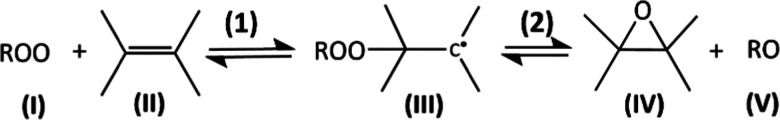
Mechanism for the addition of RO_2_ onto unsaturated compound proposed in the literature.^16–18^

Until today, the reactions of RO_2_ with unsaturated VOCs were considered negligible at room temperature and ignored in atmospheric chemistry. This assumption was entirely based on rate coefficients obtained at high temperature (*T* ≥ 360 K) in combustion systems, which were, until recently, the only experimental data available.^16^ The combustion studies also reported epoxide formation as the sole product channel for these reactions, and proceeding by two steps illustrated in Scheme 1:^16–18^ a first and rapid addition of the RO_2_ (I) onto the double bond of the alkene (II) producing the alkyl radical (adduct) (III) (reaction (1)), followed by the slow and kinetically-limiting formation of the epoxide (IV) and alkoxy radical (V) (reaction (2)).

However, a recent kinetic study of these reactions at *T* = 298 ± 5 K for a series of RO_2_ and alkenes^19^ reported bimolecular rate coefficients, *k*^II^ (cm^3^ s^−1^) that were significantly larger than those expected from the combustion data. For instance, for CH_3_O_2_ + 2,3DM2B, *k*^II^ was reported to be ∼7 × 10^−18^ cm^3^ s^−1^ instead of 6 × 10^−20^ cm^3^ s^−1^ extrapolated from combustion data.^16^ While this previous kinetic study was based on monitoring the consumption of RO_2_^20,21^ the present work further investigates these reactions by investigating their products. For this, the reactions of 1-pentylperoxy (C_5_H_11_O_2_) with 2,3-dimethyl-2-butene (2,3DM2B) and isoprene and of methylperoxy (CH_3_O_2_) with 2,3DM2B were investigated at 300 ± 5 K by PTR-TOF-MS and GC/MS.

## Experimental

### Flow reactor experiments

**Fig. 1:**
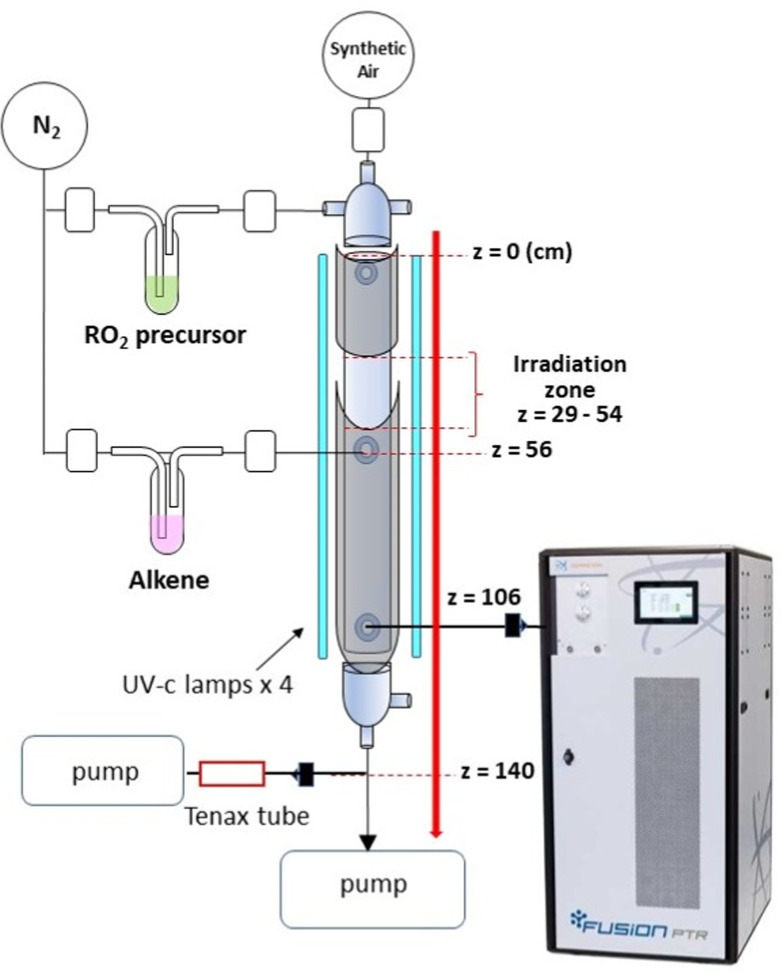
Schematics of the flow reactor set-up.

A list of the experiments and experimental conditions in this work is provided in Table S1 of the ESI.† All the experiments were conducted in a vertical Quartz reactor (length *L* = 120 cm, internal diameter *d* = 5 cm) described previously (Fig. 1).^19,21^ The bath gas was synthetic air, with flows of *F*_air_ = 1–3.5 sLm (sLm = standard temperature = 273 K and pressure = 1 atm) and operated in continuous flow, at a total pressure *P* = 0.9 atm. Under these conditions the Reynold's number is between 100 and 150, thus in the laminar regime, and the mixing length between 15 and 25 cm. Since the reactions took place beyond 50 cm in the reactor (between *z* = 56 and 106 cm) the reagents were well mixed in the experiments. The measurements were also made over timescales of ∼10–15 min, much larger than the radial diffusion time in the reactor (∼60 s), ensuring that the concentrations were radially equilibrated.

This reactor was surrounded by 4 narrow-band UV-c lamps (Phillips TUV 36W SLV/6) emitting essentially at *λ* = 254 nm. The radical precursors (iodoalkanes) were introduced in the gas by bubbling a small flow of N_2_ through the pure liquids, followed by a dilution loop, and introduced at the top of the reactor (Fig. 1). For iodopentane, the concentration used in the experiments was in the range 1–2 ppm and for iodomethane, 10–30 ppm (Table S1, ESI†). As explained in the Chemicals section below, these concentrations were determined from the ratio of the precursor and total flows in the reactor. In this study, the radicals were produced in the upper part of the reactor by irradiating a 25 cm-section above *z* = 54 cm (Fig. 1), the remainder of the reactor being kept in the dark with aluminum foil. The alkenes were also introduced in the gas by bubbling a small flow of N_2_ through the pure compounds followed by a dilution loop, but were injected below the irradiation zone (*z* = 56 cm) (Fig. 1). Therefore, the reactions RO_2_ + alkene took place entirely in the dark in our experiments, while the RO_2_ were produced photolytically in the upper part of the reactor. The alkene concentrations used in the experiments were 1–10 ppm for 2,3DM2B and 1–5 ppm for isoprene. These concentrations were limited by the upper limit of detection of the Proton Transfer Reaction Time-of-Flight Mass Spectrometry (PTR-ToF-MS) instrument used in this study. A fraction of the total flow (∼350 sccm) was sampled for PTR-ToF-MS analysis (see details below) at *z* = 106 cm (Fig. 1). In order to apply first-order kinetic analysis to the results, the experiments were performed at two different flow regimes, typically 3 and 1 sLm, so that the sampling at *z* = 106 cm corresponded to reaction times of 17 s and 53 s, respectively. The concentrations of iodoalkane and alkene and the total reactor pressure were kept identical in both regimes by adjusting the flows accordingly. The iodoalkanes and alkenes were flown continuously through the reactor, the lights being switched on and off in 10–20 min cycles to produce the RO_2_ and trigger the reactions. This was done in order to distinguish the actual reaction products from pollution in the reactor or impurities introduced together with the gas precursors. Because the alkene concentrations that could be used were limited, the RO_2_ self-reactions were significantly competing with RO_2_ + alkene reactions in the experiments. In each series of experiment, at least one cycle was performed with RO_2_ being produced while [alkene] = 0 to examine the impact of the RO_2_ self-chemistry alone.

The temperature inside the reactor was determined in separate series of experiments with an infrared thermometer (Extech 101) to 300 ± 5 K, where the uncertainties include both the variabilities in each experiment and over the time span of the study.

### Radical generation

All the RO_2_ were produced by irradiating the corresponding iodoalkane at 254 nm with the UV-c lamps. For CH_3_O_2_ the precursor was iodomethane, CH_3_I, which reacted as:

1 CH_3_l + *hν* → CH_3_ + I,

2 CH_3_ + O_2_ + M → CH_3_O_2_ + M.

For 1-pentylperoxy, C_5_H_11_O_2_, it was 1-iodopentane, C_5_H_11_I, which reacted as:

3 C_5_H_11_I + *hν* → C_5_H_11_ + I,

4 C_5_H_11_ + O_2_ + M → C_5_H_11_O_2_ + M.

Separate series of experiments showed that, over the 25 cm window, 18% of C_5_H_11_I was photolyzed in a total flow of 3 sLm (∼8 s of residence time in the irradiated zone) and 40% in a flow of 1 sLm (∼26 s of residence time), corresponding to a photolysis rate of 0.02 s^−1^. CH_3_I was assumed to have the same photolysis rate, as both compounds have identical UV absorption spectra.^22^ The concentrations of RO_2_ just above alkene injection (*z* = 54 cm in Fig. 1), [C_5_H_11_O_2_]_0_ and [CH_3_O_2_]_0_, were then estimated by kinetic modeling with ChemSimul (V3.90), taking into account the photolytic rates, self-reactions and reaction of RO_2_ with HO_2_, and autoxidation reactions in the case of C_5_H_11_O_2_.^21^ Initial concentrations of C_5_H_11_I of 3.4 and 6.0 × 10^13^ cm^−3^ (Table S1, ESI†) gave [C_5_H_11_O_2_]_0_ = 1 to 2.5 × 10^12^ cm^−3^, and initial concentrations of CH_3_I of 2.3 to 7.7 × 10^14^ cm^−3^ gave [CH_3_O_2_]_0_ = 1.5 to 3.5 × 10^12^ cm^−3^.

**Fig. 2:**
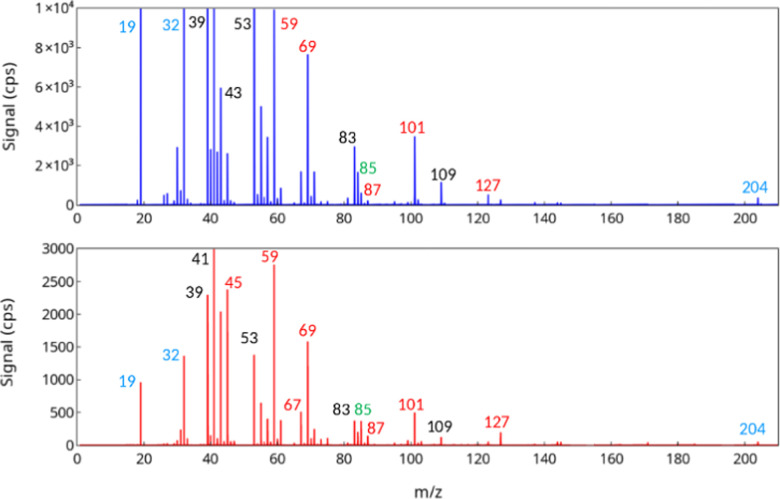
Typical mass spectra in the investigation of C_5_H_11_O_2_ + 2,3DM2B (experiment Alk38, highest peaks truncated to focus on the reaction products): Top: Overall mass spectrum obtained by averaging 16 spectra; Bottom: Differential mass spectrum. Signals labelled in blue are ions produced by the source (H_3_O^+^, *m/z* = 19. 018; O_2_^+^, *m/z* = 31.989) or internal calibrant (di-iodobenzene fragment, *m/z* = 203.943); in green are the protonated reagents (see text); signals labelled in black are attributed to fragment ions from the reagents (mostly 2,3DM2B); those labelled in red are attributed to reaction products.

Potential side-chemistry from the iodine atoms did not seem to impact the experiments. The fastest expected reactions would be I + alkene, for which the rate coefficients at 300 K are estimated to ∼10^−17^ cm^3^ s^−1^.^23^ I-Atom concentration was modeled to be of the order of 10^13^ cm^−3^ in the irradiation zone, thus consuming the alkene at rates of ∼10^−4^ s^−1^, thus slower than the RO_2_ + alkene reactions (Fig. 2). This and other I-atom reactions should produce iodinated compounds, which were found to be only minor among the observed ions (as explained below, at the resolution used in the experiments, the observed *m/z* allowed to distinguish between iodinated ions and C_*x*_H_*y*_O_*z*_^+^ ones). Most of the produced I atoms seemed either to remain unreacted or to recombine into I_2_ in the reactor, as shown by the ions at *m/z* = 126.90 and 253.81 observed at the bottom of the reactor (Tables S2–S4, ESI†).

### Product analysis by PTR-ToF-MS

The reaction mixtures were analyzed in real time by PTR-ToF-MS using a state-of-the-art FUSION PTR-TOF 10k (Ionicon Analytik Gmbh, Innsbruck, Austria).^24^ This instrument will be fully described and characterized elsewhere. Briefly, it consists of an orthogonal-acceleration reflectron Time-of-flight, allowing for a mass resolution up to 10 000, a drift tube (where the proton-analyte reactions take place) equipped with RF-ring-electrodes and ion funnel combination, an inlet system injecting the sample directly in the axis of the drift tube, and a TRION source, allowing to generate either H_3_O^+^, NO^+^, NH_4_^+^ or O_2_^+^ reagent ions. In this study, only H_3_O^+^ ions were used. The organic compound, “A”, present in the reaction mixtures were thus ionized by proton exchange with H_3_O^+^:

5 H_3_O^+^ +A → AH^+^ + H_2_O.

The drift tube was operated around 3.8 mbar and with a voltage of ∼250 V, corresponding to an DC field of strength *E/N* ∼ 40 Td (1 Td = 10^−21^ V m^2^). RF fields were also applied to the drift tube, which added some substantial energy.

The data were analyzed with the PTR-MS Viewer software V3.4.3.12 (Ionicon Analytik Gmbh, Innsbruck, Austria). After determination of the elemental composition of the ion signals (Tables S2–S4, ESI†), these were tentatively attributed to either fragment ions from the reagents or to reaction products based on their time trace. Note that, with a mass resolution of 7000–9000, it was possible to attribute a single sum formula to all ions with *m/z* ≤ 250, thus all those discussed in this work. In particular, it was possible to distinguish iodine-containing ions from C_*x*_H_*y*_O_*z*_^+^ ions because of the large negative mass defect of iodine. The search for isomers was made using the MOLGEN software online.^25^

### Tenax sampling and analysis by gas chromatography/electron impact mass spectrometry

To confirm the attribution of some of the ions observed by PTR-ToF-MS to specific isomers, samples of reaction mixtures in C_5_H_11_O_2_ + 2,3DM2B experiments (Alk39 and Alk40, Table S1, ESI†) were collected on Tenax sorbent tubes (porous polymer based on 2,6-diphenyl-*p*-phenylene oxide) for Gas Chromatographic/Electron Impact Mass Spectrometric (GC/EIMS) analysis. These experiments were performed in the flow reactor, identically to the other experiments, and five Tenax samples were collected at the outlet of the reactor, *z* ∼ 140 cm (Fig. 1) corresponding to reaction times of 19 and 60 s for flows of 3 and 1 sLm, respectively: the first sample (referred to as “sample 3” in both experiments) was taken in the dark, before the reaction took place, but with iodopentane and 2,3DM2B flowing into the reactor. The four other samples were taken during experiments, samples 4 and 5 from a total flow of 3 sLm, and samples 6 and 7 from a flow of 1 sLm. In each flow regime the 2,3DM2B concentration was varied: for samples 4 and 6 it was 2.7 × 10^14^ cm^−3^ and in sample 5 and 7, 7.2 × 10^13^ cm^−3^ (Table S1, ESI†). The samples were collected on the Tenax tubes for 1 h with a sampling flow between 30 and 40 sccm then desorbed in a Gas Chromatograph (Agilent Technologies 7890A) equipped with a Thermal Desorption Unit (Gerstel), a HP-5MS column (5% Phenyl Methyl Siloxane, 30 m × 0.25 mm × 0.25 μm), and helium as carrier gas. The chromatograph was connected to a Mass Spectrometer with triple axis detector (Agilent Technologies 5975 C) using electron ionization as ionization technique. The TDU program had an initial temperature of 120 °C, maintained for 1 min before to be ramped up to 250 °C over 11 s, and maintained for 5 min. The GC oven program had an initial temperature of 30 °C, which was maintained for 13 min. It was then ramped up to 100 °C over 7 min, maintained for 30 s, then ramped up again to 240 °C over 6.5 min and maintained for 1.5 min.

### NMR analyses

^1^H-NMR analyses of the bulk 2,3DM2B and isoprene employed in the experiments were performed with a 400 MHz Bruker Ascend. The samples were placed in CDCl_3_ and 128 scans were taken (10 min).

### Chemicals

Gases: synthetic air HiQ 5.0 (≥99.999%), N_2_ HiQ 5.5 (≥99.9995), all Linde Gas. Liquids: iodomethane, 99.5%, stab. with copper, Alfa Aesar AB; iodopentane, 97%, stabilized, Thermo Scientific; isoprene, 99%, stabilized with ∼0.02% 4-*tert*-butylcathechol, Alfa Aesar; 2,3-dimethyl-2-butene, 98%, Acros Organics; *cis*-2,3-epoxybutane, 97%, thermo scientific. The alkenes and iodoalkanes were placed in glass bubblers and introduced in the reactor by sending controlled flows of N_2_ through the liquids, followed by a dilution loop. The gas-phase concentration of these compounds in the reactor were determined from the ratio of their flows to the total flow and from their vapor pressure at 300 K (in cm^−3^): *Pv*(CH_3_I) = 1.3 × 10^19^; *Pv*(C_5_H_11_I) = 1.4 × 10^17^; *Pv*(isoprene) = 1.8 × 10^19^; *Pv*(2,3DM2B) = 4.0 × 10^18^.

## Results

### Ion attribution (overall mass spectra)

The experiments proceeded by flowing the precursors (alkenes and iodoalkanes) continuously through the reactor, and periodically switching the UV lights ON and OFF in the irradiation window to produce the RO_2_. As explained in the Experimental section, the RO_2_ were generated in the upper part of the reactor, while their reactions with alkenes took place entirely in the dark in the lower part. The experiments then proceeded in two parts, the first one corresponding to measurements performed at a reaction time of 17 s, the second to a reaction time of 53 s. In each part, the alkene concentration was gradually decreased, starting by the largest and following the values given in Table S1 (ESI†). An example of overall mass spectrum obtained with PTR-TOF-MS in the presence of RO_2_ is shown in Fig. 2. These spectra were dominated by the peaks from the reaction precursors, mostly the alkenes and their ion fragments: 2,3DM2B (C_6_H_12_) at *m/z* = 85.101 (C_6_H_12_H^+^), isoprene (C_5_H_8_) at *m/z* = 69.070 (C_5_H_8_H^+^) and 137.132 ((C_5_H_8_)_2_H^+^) and their fragments at *m*/*z* = 39.023, 41.039, 43.054, 53.002 … (in black in Fig. 2) (Tables S2–S4, ESI†). These large peaks did not display any significant variations between the RO_2_ “ON” and RO_2_ “OFF” cycles thus indicating that they were not reaction products. Ions corresponding to the iodoalkanes were also detected, but with much lower intensities, at *m/z* = 142.935 (CH_3_IH^+^) for CH_3_I, and *m/z* = 198.998 (C_5_H_11_IH^+^) for C_5_H_11_I.

### Identification of the reaction products

**Fig. 3:**
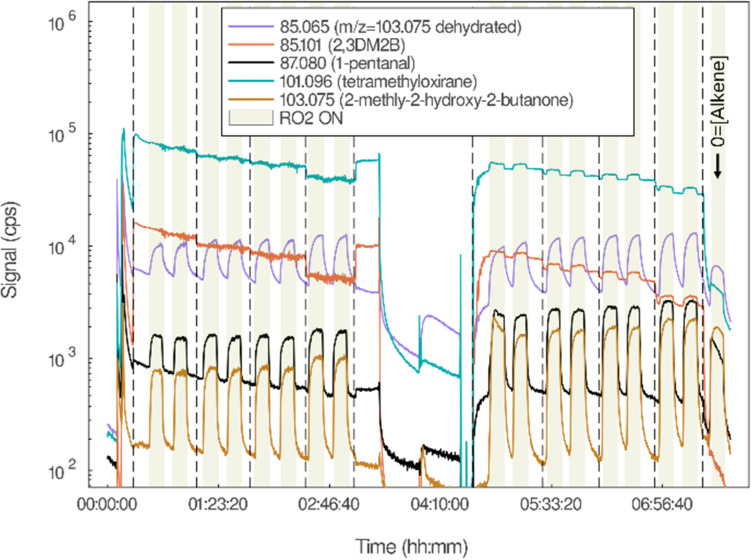
Time-evolution of the ion signals observed with FUSION PTR-ToF-MS in the investigation of C_5_H_11_O_2_ + 2,3DM2B (experiment Alk38). The shadowed areas correspond to RO_2_ “ON” cycles, the last cycle being performed with [2,3DM2B] = 0. The dashed lines indicate changes in [2,3DM2B]. The PTR-MS measurements were interrupted in the middle of the experiments to take Tenax samples.

Potential reaction products were searched among the ions displaying a significant increase in intensity in the presence of RO_2_. For this, differential mass spectra were established by subtracting the average spectrum obtained with RO_2_ “ON” from the one obtained with RO_2_ “OFF”. An example is shown in Fig. 2. However, not all peaks in these differential spectra necessarily corresponded to reaction products. In fact, the most intense ones were residual signals from the subtraction of the largest peaks of the overall spectra. Thus, a first criterion to identify reaction products, either from RO_2_ + alkene or from RO_2_ side-chemistry, was to verify that their time-profiles displayed some systematic increase during the RO_2_ “ON” cycles (see examples in Fig. 3). Next, these products were attributed either to RO_2_ + alkene reactions or to RO_2_ side-chemistry (self-reaction, autoxidation…) depending on whether they were produced in the absence of alkene and on whether their time-profiles varied proportionally or inversely with alkene concentration during the reactions. Complete lists of the ions observed in the differential spectra and their attributions are given in Tables S2–S4 (ESI†).

### Evidence for RO_2_ + alkene reaction

In all the reactions studied, the aldehyde corresponding to the RO_2_ (I) was expected to be one of the main products, as it should have been produced both by RO_2_ + alkene (from the alkoxy radical (V) in Scheme 1) and by the RO_2_ self-reaction. In the experiments with C_5_H_11_O_2_ an intense signal at *m/z* = 87.080, corresponding to the protonated ion of 1-pentanal (C_5_H_10_OH^+^), was indeed observed and displayed large variations between the RO_2_ “ON” and RO_2_ “OFF” cycles (Fig. 3 and Fig. S5a, ESI†). In the experiments with CH_3_O_2_, an intense ion signal was observed at *m/z* = 31.018 (CH_2_OH^+^), corresponding to the protonated ion of formaldehyde, CH_2_O (Table S4, ESI†), which also varied significantly between RO_2_ “ON” and RO_2_ “OFF” (Fig. S5, ESI†). However, the concentration of these aldehydes was found to vary inversely with that of the alkene during the reactions and to be maximum in the absence of alkene (profiles in black in Fig. 3 and Fig. S5 (ESI†), where the alkene concentration is gradually decreased during the experiment). In addition to the time-profiles, plots of the signal intensity for these ions as function of alkene concentration are provided in Fig. S5e–g (ESI†) and evidence these trends. This indicated that most, if not all, of these aldehydes were produced by the RO_2_ self-reaction rather than by RO_2_ + alkene. The fact that aldehyde formation decreased markedly with alkene concentration demonstrated that the RO_2_ self-reaction was in competition with RO_2_ + alkene, and thereby confirmed that the latter was taking place in the experiments.

Because this trend in aldehyde formation was important to understand the reactions in the experiments, it was confirmed by GC/MS analysis for the reaction C_5_H_11_O_2_ + 2,3DM2B. Since the ion at *m/z* = 87.080 corresponds to 74 isomers^25^ it was necessary to ensure that the trends observed with PTR-TOF-MS were specifically those of 1-pentanal and not of one of its isomers (such analysis was not necessary for the reactions of CH_3_O_2_, since formaldehyde, CH_2_O, has only one isomer). In the gas chromatograms, 1-pentanal was identified at its retention time of 4.67 min (Fig. S6, ESI†). Integrating the peaks for its main EIMS ions, at *m/z* = 44 and *m/z* = 85 (the later only in Expt. Alk40), provided its concentration in different samples corresponding to different alkene concentrations. The results are presented in Fig. S7a (ESI†) and show that, for a given reaction time, 1-pentanal concentration was systematically larger in the samples corresponding to the lowest alkene concentration: in sample 5 (2.9 ppm of 2,3DM2B) compared to 4 (10.8 ppm of 2,3DM2B), and in sample 7 (2.9 ppm of 2,3DM2B) compared to 6 (10.8 ppm of 2,3DM2B). This confirmed that this aldehyde was mainly produced by the self-reaction of C_5_H_11_O_2_ and that the reaction C_5_H_11_O_2_ + 2,3DM2B was taking place and efficiently competing with the self-reaction.

### Epoxide formation

Next, the formation of epoxide (IV) (Scheme 1), the main expected product of RO_2_ + alkene, was investigated. First, the detection of an epoxide standard, epoxybutane (C_4_H_8_O), was tested with PTR-ToF-MS to determine if proton transfer was efficient for this class of compounds. Unfortunately, no commercial standard was available for tetramethyloxirane, the epoxide from 2,3DM2B, nor for the isoprene epoxides, 2-methyl-2-vinyloxirane and 3-methyl-2-vinyloxirane. With epoxybutane, a single ion at *m/z* = 73.065 was observed, corresponding to C_4_H_8_OH^+^ and confirming that epoxides were efficiently detected by PTR-ToF-MS in our experiments.

In all the experiments, intense ion signals corresponding to the expected epoxides were observed. For the reactions with 2,3DM2B this ion was at *m/z* = 101.096 (C_6_H_12_OH^+^) corresponding to protonated tetramethyloxirane (or 2,3-dimethyl-2,3-epoxybutane), C_6_H_12_O (Fig. 3, Fig. S5c and Tables S2, S4, ESI†). However, the time profiles in the experiments suggested that it was not a reaction product: first, this ion was observed as soon as 2,3DM2B was injected in the reactor and before any reaction took place. Second, it hardly displayed any variations in the presence and the absence of RO_2_ (≤10% of its background signal, Fig. 3 and Fig. S5c, ESI†). Such small variations were within the uncertainties resulting from the normalization of the signals with the parent ion signal, H_3_O^+^. A signal increase of similar amplitude was also observed for this ion in the absence of alkene (see for instance Fig. S5c, ESI†), confirming that these variations resulted from normalization artefacts and not from RO_2_ + 2,3DM2B. Thus, in spite of a large “background” of tetramethyloxirane throughout the experiments, this compound did not seem to be produced by the reaction RO_2_ + 2,3DM2B.

To verify that any epoxide formation in the reaction RO_2_ + 2,3DM2B should have resulted in measurable signal variations, kinetic simulations were performed, using ChemSimul. 3.90 (Section S8, ESI†). The results show that, under our experimental conditions, C_5_H_11_O_2_ + 2,3DM2B should have produced up to 1.4 × 10^11^ cm^−3^ of tetramethyloxirane. Assuming a typical detection sensitivity for epoxides of 5000 cps per ppb (see Section S8, ESI†), this should have resulted in a signal increase of up to 28 000 cps, thus almost 10 times larger than the variations observed. Similarly, CH_3_O_2_ + 2,3DM2B should have produced up to 5 × 10^10^ cm^−3^ of tetramethyloxirane and resulted in a signal increase of up to 10 000 cps, thus 2.5 times larger than the variations observed. A production of epoxide in these reactions should thus have been measurable in our experiments.

The presence and production of tetramethyloxirane in the experiments was further investigated by GC/MS and ^1^NMR. This was necessary because the signal at *m/z* = 101.096 corresponds to 210 isomers,^25^ and it was important to verify that the minor signal variations on the PTR-ToF-MS time profiles did not result from the opposite contributions of different isomers, for instance. ^1^H-NMR analyses of the 2,3DM2B sample indicated the presence of 0.2% of tetramethyloxirane as main impurity (Fig. S9, ESI†). This was consistent with the observation of this compound as soon as 2,3DM2B was injected in the reactor and before any reaction took place. The concentration of this compound in experiments Alk39 and Alk40 was quantified with CG/MS by its retention time at 4.49 min (Fig. S6, ESI†). The results of the integration of the main EIMS ion peak at *m/z* = 59 are shown in Fig. S7b (ESI†). They confirmed that tetramethyloxirane was present in the reactor before any reaction took place (sample 3) and that its concentration did not increase during the reaction (samples 4–7). These results definitely confirmed that tetramethyloxirane was not produced by C_5_H_11_O_2_ + 2,3DM2B in our experiments.

The reaction with isoprene was expected to produce two epoxide isomers of brut formula C_5_H_8_O: 3,4-epoxy-3-methyl-1-butene (or 2-methyl-2-vinyloxirane) and 3,4-epoxy-2-methyl-1-butene (or 3-methyl-2-vinyloxirane). An intense ion signal was observed at *m/z* = 85.065 in all the C_5_H_11_O_2_ + isoprene experiments, which corresponds to the protonated epoxides, C_5_H_8_OH^+^ (Table S4, ESI†). This ion was observed in the reactor as soon as isoprene was injected and before any reactions had taken place. ^1^H-NMR analyses of the isoprene sample showed the presence of impurities, of structure consistent with the isoprene epoxides, and for a total concentration not exceeding 2% (Fig. S8, ESI†). This confirmed that these epoxides were present as impurities in the isoprene sample. Unlike in the 2,3DM2B experiments, the signal at *m/z* = 85.065 displayed some significant variations between the RO_2_ “ON” and RO_2_ “OFF” cycles (∼5000 cps and ∼40% of the background signal, Fig. S5a, ESI†). The kinetic simulations (Section S8, ESI†) showed that C_5_H_11_O_2_ + isoprene should have produced 3 × 10^10^ cm^−3^ of epoxide, corresponding to a signal increase of about 7000 cps, thus only slightly larger than those observed. However, the amplitude of these variations decreased with increasing isoprene concentrations during the reaction and were maximum with [alkene] = 0 (see Fig. S5a at *t* = 17 s, for instance, ESI†). This indicated that they resulted from an isomer of the epoxides produced by RO_2_ side-chemistry rather than by RO_2_ + isoprene. The formation of epoxide by RO_2_ + isoprene, in addition to this product, should have thus resulted in significantly larger signals than those observed. The reaction C_5_H_11_O_2_ + isoprene thus did not seem to produce any significant amounts of epoxide either.

The lack of epoxide production in RO_2_ + alkene in our experiments suggested that the epoxide channel (2) was not taking place (or was only minor) and that an alternative pathway was more favored at room temperature.

## Identification of other products from RO_2_ + alkene

### RO_2_ + 2,3DM2B experiments

The other products of RO_2_ + alkene reactions were identified in our analysis by their lack of production in the absence of alkene and by their increase proportionally with alkene concentration during the reactions. They were therefore clearly distinguished from the products of the RO_2_ side-chemistry (self-reaction, autoxidation…), which, by contrast, had maximum ion signals in the absence of alkene and varied inversely with alkene concentration in the reactions. In many cases, however, different isomers produced by these different reactions contributed to the ion signals. This resulted, for instance, in a significant signal with [alkene] = 0 even for ions that were ultimately attributed to RO_2_ + alkene. But in most cases, the products of the RO_2_ side-chemistry had a smaller contribution than those of RO_2_ + alkene, evidenced by a smaller signal with [alkene] = 0 than during the reactions, and allowing to establish that RO_2_ + alkene produced some compounds contributing to these signals.

In the experiments with 2,3DM2B, the main products of RO_2_ + 2,3DM2B thus identified were at *m/z* = 85.065 (C_5_H_8_OH^+^) and *m/z* = 103.075 (C_5_H_10_O_2_H^+^) (Fig. 3 and Fig. S5d, ESI†). Both ions were attributed to 3-hydroxy-3-methyl-2-butanone, with *m/z* = 103.075 being the parent ion and *m/z* = 85.065 its dehydrated ion (more intense than the parent ion, indicating substantial dehydration). In the experiments with CH_3_O_2_ both ions were mostly produced in the presence of 2,3DM2B and varied proportionally to its concentration (Fig. S5d, ESI†), thus identifying them as a product of RO_2_ + 2,3DM2B. The modest signals observed with [2,3DM2B] = 0 were attributed to some small contribution of ions produced by side-reactions of CH_3_O_2_.

**Fig. 4:**
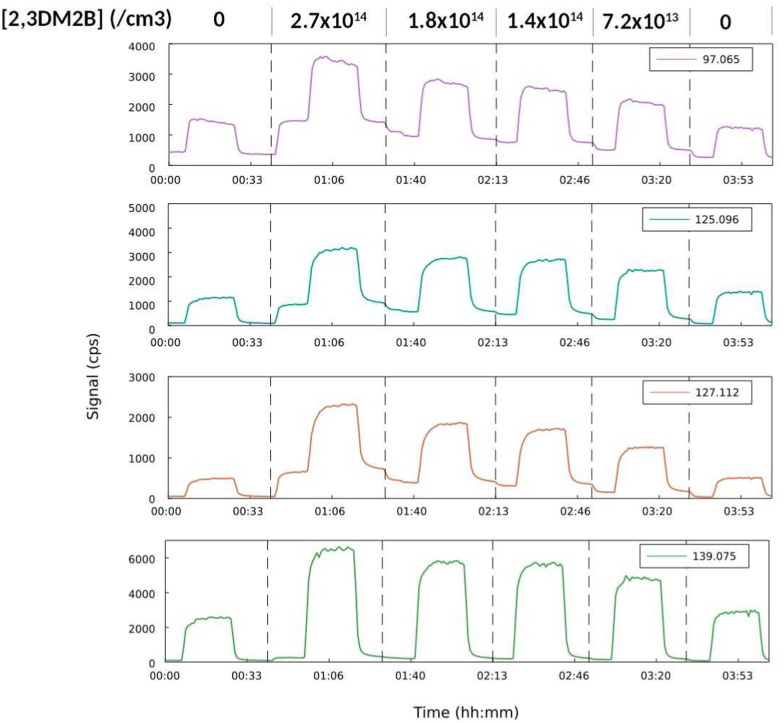
Evolution of the ion signals with [2,3DM2B] in the study of C_5_H_11_O_2_ + 2,3DM2B (experiment Alk48).

In the experiments with C_5_H_11_O_2_, these ions were also observed but their trends was complicated by a substantial contribution of pentanoic acid, an isomer of 3-hydroxy-3-methyl-2-butanone, produced by the side-reactions of C_5_H_11_O_2_ and also having an ion at *m/z* = 103.075. Other compounds clearly identified as produced by RO_2_ + 2,3DM2B were, with C_5_H_11_O_2_, *m/z* = 97.065 (C_6_H_8_OH^+^), 125.096 (C_8_H_12_OH^+^), 127.112 (C_8_H_14_OH^+^), and 139.075 (C_8_H_10_O_2_H^+^) (Fig. 4), and with CH_3_O_2_*m/z* = 97.101 (C_7_H_11_H^+^). Their variations proportional to alkene concentration is evidenced in Fig. 4, where the small ion signals in the absence of alkene were attributed to small contributions of isomers from RO_2_ side-reactions. These products could however not be identified. Finally, formaldehyde, observed at *m/z* = 31.018 (CH_2_OH^+^) and acetone, at *m/z* = 59.049 (C_3_H_6_OH^+^) were also found to be substantially produced by RO_2_ + 2,3DM2B (Tables S2–S4, ESI†). But these compounds also had other chemical sources in the experiments, in particular formaldehyde from the self-reaction of CH_3_O_2_.

All the other compounds observed during the reactions were attributed to the side-chemistry of RO_2_ (maximal formation in the absence of 2,3DM2B and variations opposite to its concentration). Those included hydroxyacetaldehyde, C_2_H_4_O_2_, observed at *m/z* = 61.028 and, mostly, at its dehydrated ion C_2_H_2_OH^+^ (*m/z* = 43.018). Ion fragments potentially resulting from autoxidation products of C_5_H_11_O_2_ were also observed such as C_5_H_8_O_2_H^+^ at *m/z* = 101.060, attributed to the dehydrated ions of the ketone-hydroperoxides C_5_H_10_O_3_ resulting from H-shift reactions in C_5_H_11_O_2_. Ions corresponding to the further dehydration and fragmentation of C_5_H_8_O_2_H^+^ were also observed, such as C_5_H_6_OH^+^ at *m/z* = 83.049 and C_4_H_6_OH^+^ at *m/z* = 71.049. In the experiments with CH_3_O_2_ much fewer ions were produced by the side-chemistry of CH_3_O_2_.

The chromatograms obtained in the GC/MS analysis of reaction mixtures of C_5_H_11_O_2_ + 2,3DM2B were also investigated for other products than the epoxide. For this, differential chromatograms were established by subtracting the chromatograms obtained with RO_2_ “OFF” (sample 3) from those obtained with RO_2_ “ON” under the same conditions (sample 4) (Fig. S11, ESI†). A number of peaks were visible in these differential chromatograms at retention times 5.5, 12.0 and 22.0 min. In particular, the product at *t* = 5.5 min was attributed to 3-hydroxy-3-methyl-2-butanone based on the mass spectra at this retention time. This was consistent with the observation of this compound as main reaction product of RO_2_ + 2,3DM2B at *m/z* = 103.076 and 85.065 with PTR-ToF-MS.

### RO_2_ + isoprene experiments

In the C_5_H_11_O_2_ + isoprene experiments, the most intense product signals were at *m/z* = 101.060 (C_5_H_8_O_2_H^+^), *m/z* = 83.049 (C_5_H_6_OH^+^), and *m/z* = 71.049 (C_4_H_6_OH^+^) (Fig. S5b, ESI†). Because these ions are isomers of those produced by the side-chemistry of C_5_H_11_O_2_ and discussed above, they were significantly produced in the absence of isoprene. However, their overall time profiles did not display any trend with isoprene concentration during the reactions, indicating that C_5_H_11_O_2_ + isoprene was producing other compounds at these masses that compensated for the negative trends from the products of the side-chemistry of C_5_H_11_O_2_. The ion at *m/z* = 101.060 (C_5_H_8_O_2_H^+^) was attributed to 2-hydroxy-2-methyl-3-butenal and *m/z* = 83.049 to its dehydrated ion, C_5_H_6_O_2_H^+^. The ion at *m/z* = 71.049 (C_4_H_6_OH^+^) was attributed to methacrolein (“MACR”) or methyl vinyl ketone (“MVK”). The latter seemed modestly produced in RO_2_ + isoprene, as most of the signal intensity was due to a large background.^26^ Weak ion signals were also observed at *m/z* = 117.055 (C_5_H_8_O_3_H^+^) (Fig. S5b, ESI†) and attributed to a small production of hydroperoxy aldehyde (“HPALD”) in RO_2_+ isoprene.^26^ Finally, the ions at *m/z* = 119.034 (C_4_H_6_O_4_H^+^) and 123.117 (C_9_H_14_H^+^) (Table S3, ESI†) were also unambiguously identified as products of RO_2_ + isoprene. But they contributed to weaker signals and could not be identified (Fig. S5b, ESI†).

## Discussion

The products reported above for the RO_2_ + alkene reactions can not be accounted for by the epoxide channel. 3-Hydroxy-3-methyl-2-butanone, acetone, 2-hydroxy-2-methyl-3-butenal, and methyl vinyl ketone (or methacrolein) have branched structures and can not be explained for by further reactions of the linear-chain alkoxy radicals from 1-C_5_H_11_O_2_ or CH_3_O_2_ (1-C_5_H_11_O and CH_3_O) co-produced in the epoxy channel. These products retain some of the alkene structure and appear to replace the expected formation of epoxide. They can not be explained by further reaction of the epoxides either, as those are rather slow in the gas phase for epoxides not containing –OH substituents (*k*^II^_OH+epoxide_ ≤ 6 × 10^−12^ cm^3^ s^−1^).^27,28^ Furthermore, the large alkene concentrations in our experiments ensured that OH radicals (as well as most other types of oxidants) were nearly inexistant. Therefore, the production of these compounds by the RO_2_ + alkene reactions implies that an alternative pathway to epoxide formation must be taking place at room temperature.

### Epoxide channel *vs.* peroxy radical channel: proposed mechanism

**Scheme 2:**
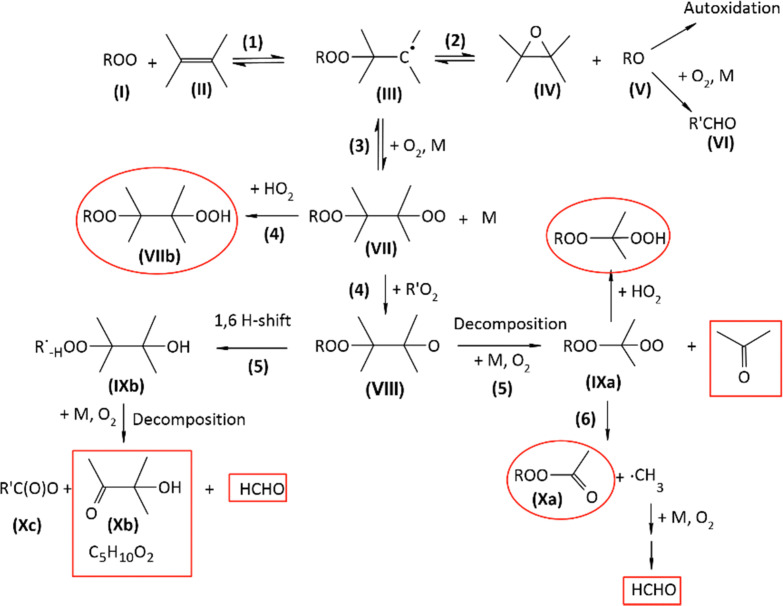
Proposed mechanism for the reactions of RO_2_ with 2,3DM2B. Red boxes are the products observed in the experiments, and red circles those expected but not observed.

**Scheme 3:**
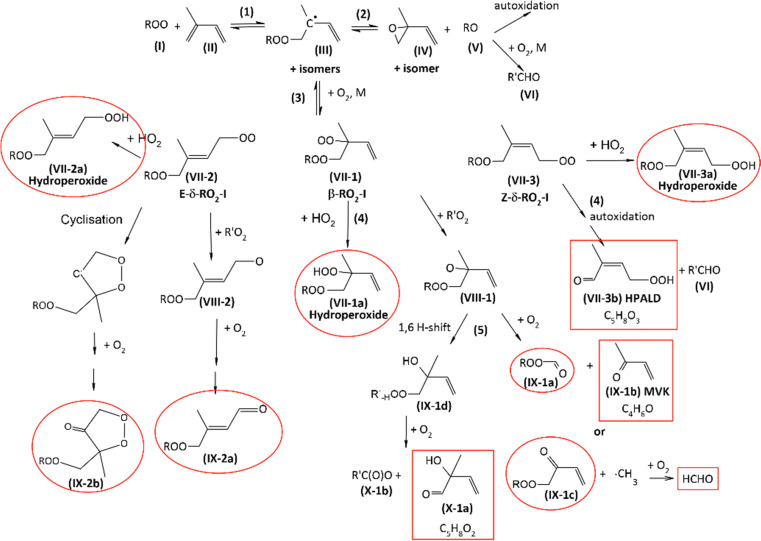
Proposed mechanism for the reaction of RO_2_ with isoprene. For clarity, only the addition on the first carbon atom of isoprene is represented. The red boxes are the products observed in the experiments and the red circles those expected but not observed.

The mechanisms proposed for the reactions of RO_2_ with 2,3DM2B and isoprene are presented in Schemes 2 and 3, respectively. We suggest that, at room temperature, instead of producing the epoxide (IV), the alkyl radical (III) reacts with O_2_ and produces a peroxy radical (VII) (Schemes 2 and 3). The peroxy radical (VII) can then undergo a series of reactions, depending on its structure and the reaction conditions. The mechanisms of these reactions are generally similar to those following the addition of OH radical on these alkenes, except that the presence of the –OOR group instead of an –OH group allows for some additional isomerizations (H-shift reactions), thus reaction products.

### Reactions with 2,3DM2B

In the reaction RO_2_ + 2,3DM2B (Scheme 2) the peroxy radicals (VII) would either cross-react with the initial peroxy radical (I) to produce a highly substituted alkoxy radical (VIII) or react with HO_2_ to produce the hydroperoxide (VIIb), although no hydroperoxide were observed in the experiments. H-shift reactions in (VII) are expected to be slow (≤10^−4^ s^−1^).^12^ Similarly to the reaction of 2,3DM2B with OH,^29^ most of the alkoxy radical (VIII) is expected to decompose into acetone. But this time the co-product is a substituted alkyl radical leading, after reaction with O_2_ to the peroxy radical (IXa), then to the peroxide-ketone (Xa) and formaldehyde. The observation of acetone in the reactions with 2,3DM2B in our experiments confirms that this pathway took place, although the peroxide (Xa) was not observed. Alternatively, the radical (IXa) can react with HO_2_ to produce an hydroperoxide. Another possible reaction pathway for the alkoxy radical (VIII), not allowed in the reaction of 2,3DM2B with OH, would be a 1,6 H-shift producing the alkyl radical (IXb). The recombination of the alkyl radical (IXb) with O_2_ would lead to a peroxy radical group directly adjacent to the peroxide group, likely to decompose into 2-methyl-2-hydroxy-2-butanone (Xb), the organic acid (Xc), and formaldehyde. The observation of (Xb) as main product of this reactions in the experiments supports the occurrence of this reaction pathway. Apart from the lack of detection of the organic peroxides (Xa) and hydroperoxides (due to ionization problems in PTR-MS, see discussion below), this mechanism is consistent with the products observed in the experiments.

### Reaction with isoprene

The mechanism of the reaction with isoprene (Scheme 3) is also similar to that of the reaction isoprene + OH. Because the latter is very complex and has been extensively studied in the literature, this discussion will focus on the main features and the on the steps that differ from the reaction with OH. As for OH radical, the initial peroxy radical (I) can add on either one of the double bonds leading, after allylic rearrangement, to six possible alkyl radicals (III).^26^ Only the major pathways, corresponding to the addition of (I) on the first carbon atom and leading to the most substituted alkyl radical (III) will be further discussed and illustrated in Scheme 3. Note that the other pathways would produce compounds that are either identical to or isomers of those described below, and would not be differentiated from them with PTR-ToF-MS. We propose that, instead of producing an epoxide, (III) recombines with molecular oxygen. This produces three isomer peroxy radical, (VII-1), (VII-2), and (VII-3), referred to as β-RO_2_-I, *E*-δ-RO_2_-I, and *Z*-δ-RO_2_-I, respectively, by analogy with the isoprene oxidation mechanisms proposed in the literature.^14,26,30^ All three radicals could react with HO_2_ to produce the hydroperoxides (VII-1a), (VII-2a), and (VII-3a). In addition, (VII-1) and (VII-2) can cross-react with other RO_2_ to produce the alkoxy radicals (VIII-1) and (VIII-2). For (VII-3) (*Z*-δ-RO2-I), however, H-shift reactions are fast and would lead ultimately to the hydroperoxyl aldehyde (HPALD) (VII-3b),^14,26^ observed as a minor product in our experiments. In addition, (VII-2) (*E*-δ-RO_2_-I) can possibly undergo cyclisation to lead ultimately to the cyclic organic peroxide (IX-2b). Further reactions of the alkoxy radical (VIII-1) with molecular oxygen would produce methyl vinyl ketone (MVK, IX-1a), observed in our experiments, and the peroxide aldehyde (IX-1b) or, alternatively the peroxide ketone (IX-1c) and formaldehyde. The alkoxy radical (VIII-1) could also undergo a 1,6H-shift reaction, leading to the alkyl radical (IX-1d) and ultimately to 2-hydroxy-2-methyl-3-butenal (X-1a) and the organic acid (X-1b). The observation of (X-1a) with PTR-ToF-MS suggests that this reaction pathway is significant. Finally, further reactions of the alkoxy radical (VIII-2) with oxygen should lead ultimately to the peroxide ketone (IX-2a).

Thus, while not all the product expected from these mechanisms were observed in our experiments, those observed support the main proposed pathways rather than the formation of epoxide. In all the experiments, the products that were not detected were systematically those containing either a hydroperoxide or an organic peroxide structure. This is likely due to fragmentation in the ionization of these compounds with PTR-MS, as recently reported.^31^ To verify this, we attempted the detection of a standard mixture of *t*-butyl peroxide (99% Thermo Scientific, 300 ppb in N_2_) with PTR-ToF-MS. No signal was observed at the expected *m/z* = 147.139, nor anywhere in the spectra, confirming that organic peroxides (and probably hydroperoxides) can not be detected with PTR-MS.

### Epoxide channel *vs.* peroxy radical channel: kinetic discussion

The kinetic data available in the literature further supports our proposition that, under atmospheric conditions, the formation of the peroxy radical (VII) should be favored over that of the epoxide (IV) in RO_2_ + alkene reactions. The rate of recombination of the alkyl radical (III) with molecular oxygen (reaction (3)) is very fast: using the rate coefficient for *t*-butyl radical, *k*^II^(*t*-butyl + O_2_) = 7.5 × 10^−12^ cm^3^ s^−1^,^32^ and [O_2_] = 5 × 10^18^ cm^−3^ gives *r*_3_ ∼ 4 × 10^7^ s^−1^. Monomolecular rate coefficients for epoxidation reactions (reaction (2)) have been estimated for organic peroxides^33^ to *r*_2_ = 2 × 10^2^–5 × 10^4^ s^−1^, thus significantly slower than the reaction of the alkyl radical (III) with O_2_. According to this previous study, epoxidation rates are fast only for HO-substituted hydroperoxides.^33^ Note that, in combustion systems, this peroxy radical pathway had been ruled out as negligible compared to the epoxide channel.^17^ The rate of the epoxidation step (2) in combustion systems must have also been much smaller than estimated by ref. 33, as it was systematically reported to be kinetically-limiting over step (1).^16–18^

The fact that RO_2_ + alkene proceeds by a peroxy radical pathway instead of the epoxide pathway also implies that, at room temperature, the overall reaction is kinetically limited by the initial addition step (1) rather than the epoxidation step (2) as reported until now.^16–18^ Typical values of *r*_1_ for step 1 can be estimated from the rate coefficients recently measured at room temperature for these reactions. These measurements were based on monitoring the consumption of the RO_2_ (I), thus actually monitored the first addition step. These were *k*^II^_1_(CH_3_O_2_ + 2,3DM2B) = 6.7 × 10^−18^ cm^3^ s^−1^ and *k*^II^_1_(C_5_H_11_O_2_ + 2,3DM2B) = 1.6 × 10^−16^ cm^3^ s^−1^.^19^ The maximum concentration of [2,3DM2B] used in each type of experiment (2.7–3.5 × 10^14^ cm^−3^) thus gives *r*_1_ ≤ 2 × 10^−3^–4 × 10^−2^ s^−1^. The reactions following the formation of the peroxy radical (VI) would be faster than *r*_1_ as they involve reactions of peroxy radicals with other RO_2_ (>5 × 10^−2^ s^−1^ in our experiments), or with HO_2_ or NO in the atmosphere, and reactions of alkoxy radicals, which are also fast (>10^5^ s^−1^ for isomerisation, >10^7^ s^−1^ for recombination with oxygen). Step (1) is thus expected to be the kinetically-limiting step in most systems under atmospheric conditions.

Since the reactions RO_2_ + alkene are kinetically limited by step (1) at room temperature, but by step (2) at high temperature,^16^ extrapolating down the kinetic data obtained at high temperature should significantly underestimate the actual rate coefficients at room temperature. For CH_3_O_2_ + 2,3DM2B the combustion data lead to a rate coefficient of *k*^II^(CH_3_O_2_ + 2,3DM2B) ∼ 6 × 10^−20^ cm^3^ s^−1^.^16^ The rate coefficients for the two other reactions can be estimated from the combustion data to *k*^II^(C_5_H_11_O_2_ + 2,3DM2B) ∼ 3 × 10^−19^ cm^3^ s^−1^ and *k*^II^(C_5_H_11_O_2_ + isoprene) ∼ 6 × 10^−21^ cm^3^ s^−1^ by applying some correction factors to account for the larger RO_2_ (see discussion in ref. 19). By comparison, recent kinetic experiments reported rate coefficients recently these reactions at 298 K^19^ of *k*^II^(C_5_H_11_O_2_) + 2,3DM2B = 1.6 × 10^−16^ (0.3–8 × 10^−16^) cm^3^ s^−1^; *k*^II^(C_5_H_11_O_2_ + isoprene) = 7.8 × 10^−18^ (1.6–39 × 10^−18^) cm^3^ s^−1^; and *k*^II^(CH_3_O_2_ + 2,3DM2B) = 6.7 × 10^−18^ (3.4–33.5 × 10^−18^) cm^3^ mol^−1^ s^−1^. In the present work, attempts were made to measure these rate coefficients again but from the product build-up in the experiments. For this, pseudo-first order analyses were applied to the ion signals displaying a clear increase with alkene concentration (Section S10, ESI†): *m/z* = 127.112 for C_5_H_11_O_2_ + 2,3DM2B; *m/z* = 119.034 for C_5_H_11_O_2_ + isoprene, and *m/z* = 85.065 for CH_3_O_2_ + 2,3DM2B. The results are presented in Section S10 (ESI†). Unfortunately, under our experimental conditions the reactions were too slow to determine these rate coefficients accurately. However, the results were generally consistent (within a factor 3) with those reported recently. The differences of 1 to 2 orders of magnitude between the experimental results at room temperature and the combustion data show that the later are not adequate to estimate the rate coefficients for RO_2_ + alkene reactions under atmospheric conditions. Yet, these combustion data have been the reason for underestimating RO_2_ + alkene reactions under atmospheric conditions until now.

## Conclusions

Experimental investigations of RO_2_ + alkene reactions under atmospheric conditions (atmospheric pressure, [O_2_] = 5 × 10^18^ cm^−3^ and *T* = 300 ± 5 K) in this work showed that, unlike what had been proposed in the literature until now, these reactions did not produce any significant amount of epoxide. Instead, the formation of other products such as 3-hydroxy-3-methyl-2-butanone, acetone, 2-hydroxy-2-methyl-3-butenal, and methyl vinyl ketone suggested the existence of other reaction pathways. We propose that, at room temperature, the reaction of the initial alkyl radical adduct with oxygen to produce a peroxy radical is more favored than its epoxidation, which is supported by kinetic data.

The fact that RO_2_ + alkene proceeds by a peroxy radical pathway instead of the epoxide pathway at room temperature implies that it is kinetically limited by the initial addition step and not by the epoxidation, as reported until now for combustion systems. The kinetic data obtained from combustion systems thus do not represent adequately the mechanisms at room temperature. Extrapolating the rate coefficients from these data leads to significant underestimation at room temperature and has been responsible for overlooking these reactions under atmospheric conditions until now. These discrepancies also explain the unexpected large rate coefficients recently measured at room temperature for these reactions.

### Implications for the atmosphere and chamber studies

In the atmosphere and in chamber studies these reactions would only be significant under very low NO_*x*_ condition ([NO] ≤ 0.05 ppb). They would be expected to act as additional sinks for the RO_2_, thus potentially reducing the total RO_2_ concentration. However, because the R’O_2_ produced by RO_2_ + alkene do not allow for fast H-shift, as with their HO-RO_2_ analogs, these reactions are not expected to contribute to OH recycling and might even compete with it. The effects of RO_2_ + alkene reactions should be mostly significant for slow-reacting RO_2_ since their rate coefficients are likely to be small for many classes of RO_2_, in particular aliphatic ones, with *k*^II^_RO_2_+alkene_ ≤ 10^−15^ cm^3^ s^−1^. Only for RO_2_ with specific structures such as HO-, allyl-, or acyl substituents, they might be significant at room temperature (see structure–activity discussions in ref. 16 and 19). Previous kinetic measurements gave a rate coefficient of 10^−14^ cm^3^ s^−1^ for the reaction of peroxy acyl radical, CH_3_C(O)O_2_, with 2,3DM2B, which can be used as typical value for the reactions of functionalized RO_2_ with alkenes.

Thus, in regions of the atmosphere that are strongly impacted by biogenic emissions, a concentration [alkene] = 5 ppb^34^ would correspond to first-order rates of ∼10^−3^ s^−1^. For the RO_2_ present in these regions and having slower sinks, RO_2_ + alkene reactions could be competitive, thus acting as additional sinks and potentially accounting for the “missing RO_2_ sinks” reported in several studies.^5,6^

To estimate the potential effects of RO_2_ + alkene reactions in chamber experiments, simple kinetic simulations were performed and compared with the isoprene + OH experiments in ref. 14 (see details Section S12, ESI†). The kinetic system was kept as simple as possible to examine mostly the trends on the isoprene-RO_2_ (“IsopRO2”), total RO_2_ (ΣRO_2_), and OH recycling. With an initial concentration of [isoprene] = 5 ppb and a first order rate of the order of 10^−3^ s^−1^ for RO_2_ + isoprene, these reactions were expected to compete with the sinks of several isoprene-RO_2_, in particular with the 1,5-H shift discussed for β-RO_2_-I in ref. 14. This could potentially account for the overestimation of ΣRO_2_ by the models in ref. 14. To determine a lower limit for the impact of RO_2_ + isoprene in these experiments, the simulations only took onto account the RO_2_ channel for *Z*-δ-RO_2_-I, which has the fastest monomolecular sink rate (0.1–0.4 s^−1^, see Table S12.1, ESI†).^14^ In spite of the large differences in the reaction rates between this sink and RO_2_ + isoprene, the latter was found to significantly reduce ΣRO_2_ (by 10 to 50%) as well as OH recycling (Fig. S12.2, ESI†). This unexpectedly large effect was due to the efficient recycling of OH by *Z*-δ-RO_2_-I, largely controlling OH concentration. This, in turn, controlled the oxidation of isoprene and the total production of RO_2_. Thus, even a small competition of RO_2_ + isoprene with the main sink of *Z*-δ-RO_2_-I was sufficient to significantly reduce [OH] and the overall RO_2_ production. These results indicate that, in addition to providing additional sinks for slow-reacting RO_2_ and reducing ΣRO_2_, RO_2_ + alkene reactions could have more impacts than expected from their small reaction rates in chamber experiments where RO_2_ reactions affect OH concentration.

## Author contributions

BN supervised the study, performed the experiments, analyzed the data and wrote the paper. OD contributed to the analyses of the FUSION PTR-ToF data and to the figures in the manuscript. ED performed the GC/MS experiments and subsequent analyses. SK developed the GC/MS method employed in the study. ÅE supervised the GC/MS studies. FF contributed to the GC/MS analyses, NMR analyses, the discussion of the reaction mechanisms, and to the Schemes presented in the manuscript. FP and AW contributed to the analyses of the FUSION PTR-ToF data. All the authors read and commented the manuscript before submission.

## Conflicts of interest

There are no conflicts to declare.

## Supplementary Material

CP-025-D2CP05166D-s001
