# Supplementary material for: The reaction of organic peroxy radicals with unsaturated compounds controlled by a non-epoxide pathway under atmospheric conditions
Source: Phys Chem Chem Phys. 2023 Feb 23;25(11):7772–82. doi: 10.1039/d2cp05166d (PMC10015623; doi:10.1039/d2cp05166d)
Supplement: CP-025-D2CP05166D-s001 [file CP-025-D2CP05166D-s001.pdf]

Electronic Supplementary Material (ESI) for Physical Chemistry Chemical Physics.
This journal is © the Owner Societies 2023

Electronic Supplementary Information

The Reaction of Organic Peroxy Radicals with Unsaturated Compounds Controlled by a non-Epoxide Pathway under Atmospheric Conditions

By Barbara Nozière,[a] Olivier Durif,[a] Eloé Dubus,[a] Stephanie Kylington,[a] Åsa Emmer,[a] Fabienne Fache,[b] Felix Piel,[c] and Armin Wisthaler[c]

[a] KTH, Royal Institute of Technology, Department of Chemistry, 114 28 Stockholm, Sweden
[b]Université Lyon 1 and CNRS, UMR 5246, ICBMS, 69626 Villeurbanne, France
[c]University of Oslo, Department of Chemistry, 0315 Oslo, Norway

Table of Contents

Table S1: List of the experiments and experimental conditions. All the concentrations are in molecule cm.$^{-3}$............2
Table S2: Main ions in the experiments $C_5H_{11}O_2$ + 2,3 DM2B 3
Table S3: Main ions in the experiments $C_5H_{11}O_2$ + isoprene. 4
Table S4: Main ions in the experiments CH_3O_2 + 2,3DM2B. 5
Figure S5: Time-profiles and variations of the main aldehydes with alkene concentration in the experiments. 6
Figure S6: Chromatograms. 9
Figure S7: GC/MS analysis: Evolution of the concentration of 1-pentanal and tetramethyloxirane. 10
Section S8: Kinetic simulations 11
Table S8.1: Rate coefficients used in the simulations 11
Table S8.2: Results of the kinetic simulations 11
Figure S9: NMR spectra for the 2,3DM2B and isoprene samples. 13
Section S10: Kinetic analyses of the ion signals in the reactions RO_2 + alkene. 14
Figure S10B1: Evolution of the ion *m/z* = 127.112 in $C_5H_{11}O_2$ + 2,3DM2B experiment (Alk38)............14
Figure S10B2: Kinetic plot for Fig. S10B1............14
Figure S10B3: Evolution of the ion *m/z* = 119.034 in $C_5H_{11}O_2$ + isoprene experiment (Alk44). 15
Figure S10B4: Kinetic plot for Fig. S10B3............15
Figure S10B5: Evolution of the ion *m/z* = 85.065 in CH_3O_2 + 2,3DM2B experiment (Alk46). 16
Figure S10B6: Kinetic plot for Fig. S10B5............16
Figure S11: Chromatograms of potential reaction products............17
Section S12: Potential effects on RO_2+alkene reactions in chamber experiments (kinetic simulations) 18

Table S1: List of the experiments and experimental conditions**.** All the concentrations are in cm.$^{-3}$

		**$C_5H_{11}O_2$ + 2,3DMB**	
**Expt. n°**	F_{air} (sLm)	[$C_5H_{11}I$]	[2,3DMB]
**PTr-MS FUSION analyses**			
Alk35	3.5/1.75	3.4×10^{13}	2.0×10^{13} / 5.1×10^{13}
Alk36	3.5/1.0	3.4×10^{13}	2.0×10^{13} / 5.1×10^{13}
Alk37	3.0/1.0	6.0×10^{13}	7.2×10^{13}/ 1.4×10^{14}/ 1.8×10^{14} / 2.7×10^{14}
Alk38	3.0/1.0	6.0×10^{13}	7.2×10^{13}/ 1.4×10^{14}/ 1.8×10^{14} / 2.7×10^{14}
Alk48	1.0	6.0×10^{13}	7.2×10^{13}/ 1.4×10^{14}/ 1.8×10^{14} / 2.7×10^{14}
**GC/MS analyses**			
Alk39	3.0/1.0	6.0×10^{13}	sample 3: Lights OFF, 2.7×10^{14}<br>samples 4 and 6: Lights ON, 2.7×10^{14}<br>samples 5 and 7: Lights ON, 7.2×10^{13}
Alk40	3.0/1.0	6.0×10^{13}	sample 3: Lights OFF, 2.7×10^{14}<br>samples 4 and 6: Lights ON, 2.7×10^{14}<br>samples 5 and 7: Lights ON, 7.2×10^{13}
		**$C_5H_{11}O_2$, + isoprene (**PTr-MS FUSION analyses only**)**	
		[$C_5H_{11}I$]	[isoprene]
Alk41	3.5/1.0	3.4×10^{13}	2.3×10^{13}/ 4.6×10^{13}
Alk42	3.5/1.0	3.4×10^{13}	4.6×10^{13}/ 1.2×10^{14}
Alk43	3.0/1.0	3.4×10^{13}	4.3×10^{13}/ 6.5×10^{13}/ 8.6×10^{13}
Alk44	3.0/1.0	6.0×10^{13}	4.3×10^{13}/ 6.5×10^{13}/ 8.6×10^{13}
		**CH_3O_2 + 2,3DMB (**PTr-MS FUSION analyses only**)**	
		[CH_3I]	[2,3DMB]
Alk45	3.0/1.0	2.3×10^{14}	1.8×10^{14}/ 3.5×10^{14}
Alk46	3.0/1.0	7.7×10^{14}	1.4×10^{14}/ 1.8×10^{14}/ 2.7×10^{14} / 3.5×10^{14}
Alk47	3.0/1.0	5.5×10^{14}	1.4×10^{14}/ 1.8×10^{14}/ 2.7×10^{14} / 3.5×10^{14}

Table S2: Main ions in the experiments $C_5H_{11}O_2$ + 2,3 DM2B

m/z	Intensity (Baseline/Product)	Ion sum formula	Neutral sum formula	Interpretation and comments
19.018	10^5 / -	H_3O^+	H_2O	Source
31.018	10^3 / 10^4	CH_2OH^+	CH_2O	Formaldehyde, product
43.018	10^4 / $5x10^4$	$C_2H_2OH^+$	$C_2H_4O_2$	Fragment of $C_2H_4O_2H^+$
45.033	- / $5x10^4$	$C_2H_4OH^+$	C_2H_4O	Acetaldehyde, product side-chemistry
59.049	$5x10^4$ / $5x10^4$	$C_3H_6OH^+$	C_3H_6O	Acetone, 2,3DM2B product
61.028	10^4 / 10^4	$C_2H_4O_2H^+$	$C_2H_4O_2$	Acetic acid/hydroxyacetaldehyde, product
67.054	10^4 / 10^4	$C_5H_7^+$	/	Fragment, autoxidation $C_5H_{11}O_2$
69.070	10^5 / $5x10^4$	$C_5H_8H^+$	/	Fragment, autoxidation $C_5H_{11}O_2$
71.049	$5x10^3$ / 10^4	$C_4H_6OH^+$	/	Fragment, autoxidation $C_5H_{11}O_2$
83.049	$5x10^3$ / 10^4	$C_5H_6OH^+$	/	Fragment, autoxidation $C_5H_{11}O_2$
85.065	10^4 / 10^4	$C_5H_8OH^+$	$C_5H_{10}O_2$	3-hydroxy-3-methyl-2-butanone + pentanoic acid (dehydrated ion)
85.101	10^5 / $5x10^3$	$C_6H_{12}H^+$	C_6H_{12}	2,3DM2B
87.080	10^3 / $5x10^3$	$C_5H_{10}OH^+$	$C_5H_{10}O$	Pentanal
**97.065**	10^3 / 10^3	$C_6H_8OH^+$	C_6H_8O	Product $C_5H_{11}O_2$+ 2,3DM2B
99.044	$5x10^3$ / $5x10^3$	$C_5H_6O_2H^+$	$C_5H_6O_2$	Product RO_2 + 2,3DM2B
99.080	$5x10^3$ / $5x10^3$	$C_6H_{10}OH^+$	$C_6H_{10}O$	Product RO_2 + 2,3DM2B
101.060	10^3 / 10^4	$C_5H_8O_2H^+$	$C_5H_{10}O_3$	Fragment, autoxidation $C_5H_{11}O_2$
101.096	10^5 / 10^4	$C_6H_{12}OH^+$	$C_6H_{12}O$	Tetramethyl oxirane + 210 isomers
103.075	- / 10^3	$C_5H_{10}O_2H^+$	$C_5H_{10}O_2$	3-hydroxy-3-methyl-2-butanone + pentanoic acid
105.018	- / $5x10^2$	$C_3H_4O_4H^+$	/	Fragment, side-chemistry
105.033	- / 10^2	$C_7H_4OH^+$	/	Fragment, RO_2 + 2,3DM2B
**125.096**	$5x10^2$ / $5x10^3$	$C_8H_{12}OH^+$	/	Product $C_5H_{11}O_2$+ 2,3DM2B
126.9	10^3 / $5x10^3$	I+	I	Iodine atom
**127.112**	$5x10^2$ / 10^3	$C_8H_{14}OH^+$	/	Product $C_5H_{11}O_2$+ 2,3DM2B
**139.075**	10^3 / $5x10^3$	$C_8H_{10}O_2H^+$	/	Product $C_5H_{11}O_2$+ 2,3DM2B
253.82	- / $5x10^3$	I_2+	I_2	di-Iodine

Table S3: Main ions in the experiments $C_5H_{11}O_2$ + isoprene.

m/z	Intensity (Baseline/Product)	Ion sum formula	Neutral sum formula	Interpretation and comments
19.018	10^5 / -	H_3O^+	H_2O	Source
31.018	$5x10^3$ / 10^3	CH_2OH^+	CH_2O	Formaldehyde, product
43.018	$5x10^4$ / 10^4	$C_2H_2OH^+$	$C_2H_4O_2$	Fragment of $C_2H_4O_2H^+$
45.033	$5x10^3$ / 10^4	$C_2H_4OH^+$	C_2H_4O	Acetaldehyde, product side-chemistry
55.018	10^3 / $5x10^2$	$C_3H_2OH^+$	/	Fragment, side-chemistry
59.049	$5x10^4$ / $2x10^3$	$C_3H_6OH^+$	C_3H_6O	Acetone, product
61.028	$5x10^3$ / $5x10^3$	$C_2H_4O_2H^+$	$C_2H_4O_2$	Acetic acid/hydroxyacetaldehyde, product side-chemistry
69.070	10^5 / 10^4	$C_5H_8H^+$	C_5H_8	isoprene
71.049	10^4 / $5x10^3$	$C_4H_6OH^+$	C_4H_6O	MACR or MVK
73.028	10^3 / 10^3	$C_3H_4O_2H^+$	$C_3H_4O_2$	Product side-chemistry
83.049	$5x10^3$ / $5x10^3$	$C_5H_6OH^+$	$C_5H_8O_2$	2-hydroxy-2-methyl-3-butenal (dehydrated ion)
85.065	10^4 / $5x10^3$	$C_5H_8OH^+$	C_5H_8O	Isoprene epoxide
87.080	$5x10^2$ / 10^3	$C_5H_{10}OH^+$	$C_5H_{10}O$	Pentanal
101.060	10^3 / $5x10^3$	$C_5H_8O_2H^+$	$C_5H_8O_2$	2-hydroxy-2-methyl-3-butenal
103.075	10^2 / 10^3	$C_5H_{10}O_2H^+$	$C_5H_{10}O_2$	Pentanoic acid, product side-chemistry
105.018	50 / 10^2	$C_3H_4O_4H^+$	/	Product side-chemistry
107.034	50 / 10^2	$C_3H_6O_4H^+$	$C_3H_6O_4$	Product side-chemistry
111.080	10^2 / 10^2	$C_7H_{10}O_4H^+$	$C_7H_{10}O_4$	Product side-chemistry
113.023	$5x10^2$ / 10^2	$C_5H_4O_3H^+$	$C_5H_4O_3$	Product side-chemistry
115.039	10^2 / 10^2	$C_5H_6O_3H^+$	$C_5H_6O_3$	Product side-chemistry
117.055	50 / 10^2	$C_5H_8O_3H^+$	$C_5H_8O_3$	HPALD
**119.034**	50 / 10^2	$C_4H_6O_4H^+$	/	Product $C_5H_{11}O_2$+ isoprene
121.028	10^2 / 10^2	$C_7H_4O_2H^+$	$C_7H_4O_2$	Product
**123.117**	$5x10^2$ / $5x10^2$	$C_9H_{14}H^+$	/	Fragment $C_5H_{11}O_2$+ isoprene
126.9	$5x10^3$ / $5x10^3$	I+	I	Iodine atom
253.82	- / $5x10^3$	I_2+	I_2	di-Iodine

Table S4: Main ions in the experiments CH_3O_2 + 2,3DM2B.

m/z	Intensity (Baseline/Product)	Ion sum formula	Neutral sum formula	Interpretation and comments
19.018	10^5 / -	H_3O^+	H_2O	Source
31.018	$5x10^3$ / 10^4	CH_2OH^+	CH_2O	Formaldehyde, product
43.018	$5x10^4$ / $5x10^3$	$C_2H_2OH^+$	$C_2H_4O_2$	Fragment of $C_2H_4O_2H^+$
45.033	$5x10^3$ / 10^3	$C_2H_4OH^+$	C_2H_4O	Acetaldehyde, product side-chemistry
47.013	10^3 / 10^3	$CH_2O_2H^+$	CH_2O_2	Formic acid, product side-chemistry CH_3O_2
47.049	- / 10^3	$C_2H_6OH^+$	C_2H_6O	Product side-chemistry CH_3O_2
55.054	$5x10^4$ / 10^4	$C_4H_6H^+$	/	Fragment, side-chemistry
59.049	$5x10^4$ / 10^4	$C_3H_6OH^+$	C_3H_6O	Acetone, product RO_2 + 2,3DM2B
61.028	$5x10^3$ / 10^4	$C_2H_4O_2H^+$	$C_2H_4O_2$	Acetic acid/hydroxyacetaldehyde, product side-chemistry
73.065	10^3 / $5x10^2$	$C_4H_8OH^+$	C_4H_8O	Product side-chemistry
81.070	$5x10^3$ / 10^3	$C_6H_8H^+$	/	Fragment side-chemistry
83.049	$5x10^2$ / $5x10^2$	$C_5H_6OH^+$	/	Product side-chemistry
83.086	10^4 / $5x10^3$	$C_6H_{10}H^+$	/	Fragment side-chemistry
**85.065**	10^3 / 10^3	$C_5H_8OH^+$	$C_5H_{10}O_2$	2-methyl-2-hydroxy-2-butanone (dehydrated ion)
**97.101**	10^2 / 10^2	$C_7H_{11}H^+$	C_7H_{11}	Fragment CH_3O_2+ 2,3DM2B
126.9	10^5 / 10^4	I+	I	Iodine atom
253.82	10^4 / $5x10^4$	I_2+	I_2	di-Iodine

Figure S5: Time-profiles and variations of the main aldehydes with alkene concentration in the experiments.

a) and b): Time profiles for $C_5H_{11}O_2$ + isoprene (Experiments Alk44); c) and d) Time profiles for CH_3O_2 + 2,3DM2B (experiment Alk46); The shaded areas correspond to RO_2 "ON" cycles. The dashed lines indicate different alkene concentrations (decreasing during the experiments, with the values given in Table S1). In all the experiments, the products were first studied at a reaction time of *t* = 17 s (first part of the experiment), then at a reaction time of *t* =53 s (second part).e)-g) Variations of the aldehydes signal with alkene concentration.

(a)

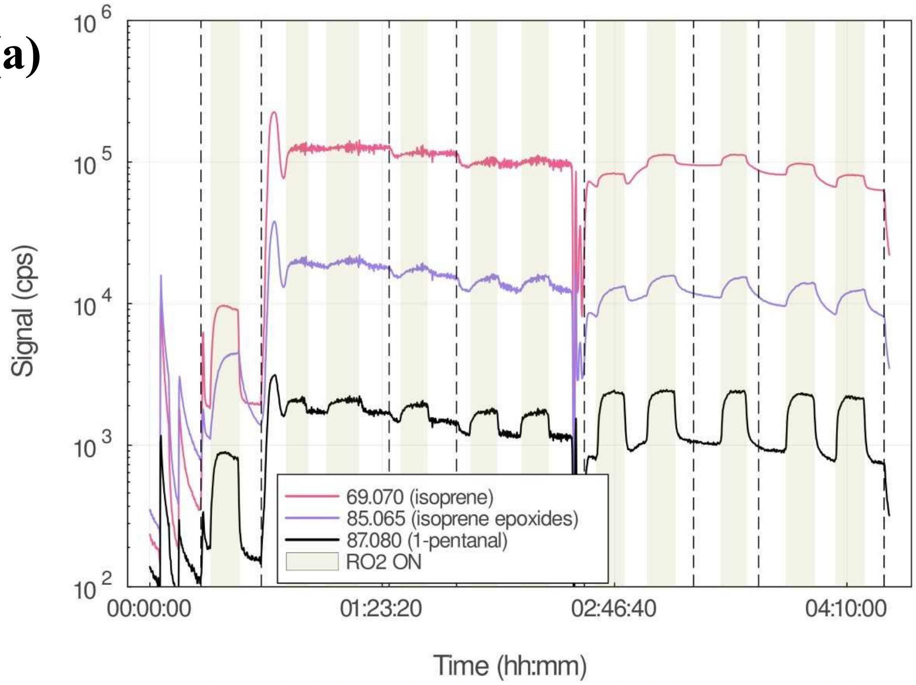

(b)

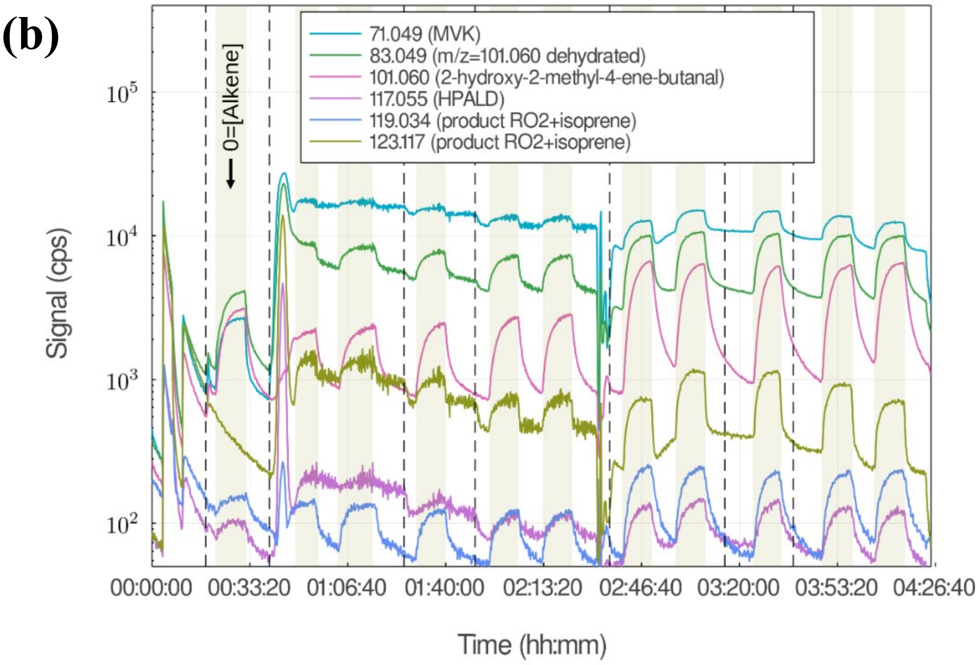

(c)

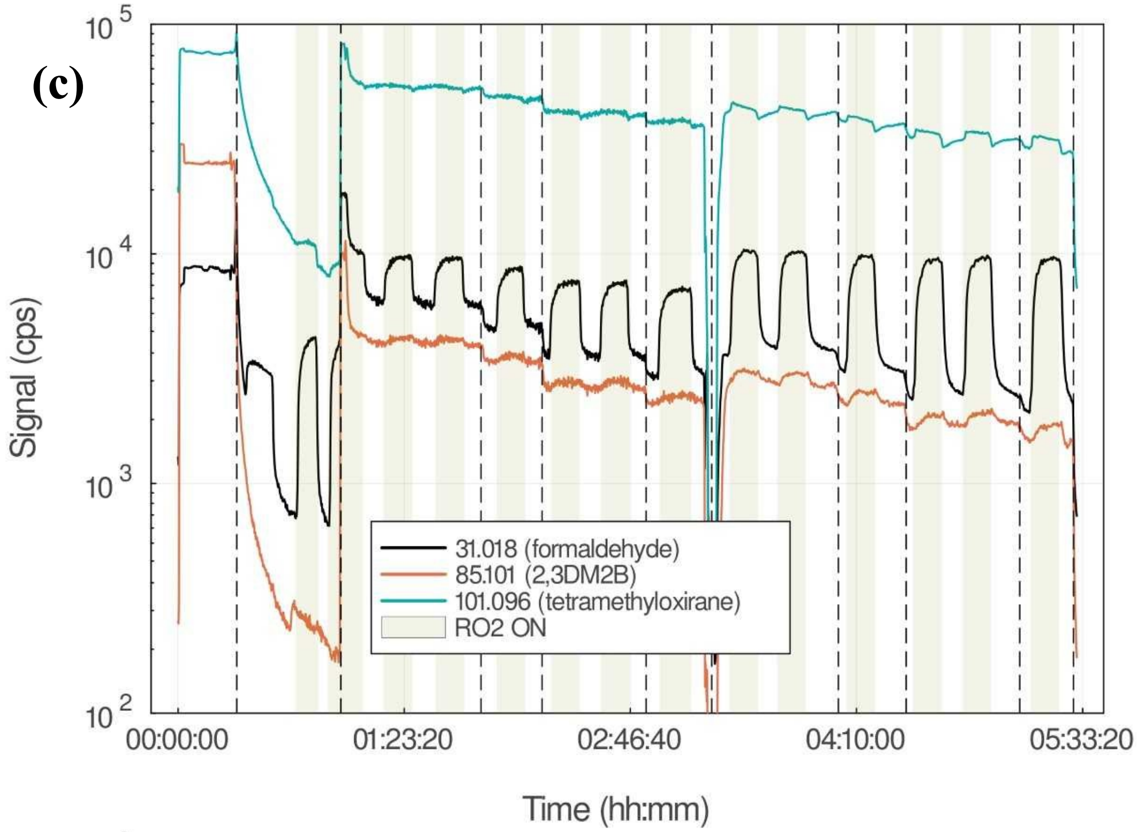

10^5
10^4
10^3
10^2
Signal (cps)
00:00:00
01:23:20
02:46:40
04:10:00
05:33:20
Time (hh:mm)
31.018 (formaldehyde)
85.101 (2,3DM2B)
101.096 (tetramethyloxirane)
RO2 ON

(d)

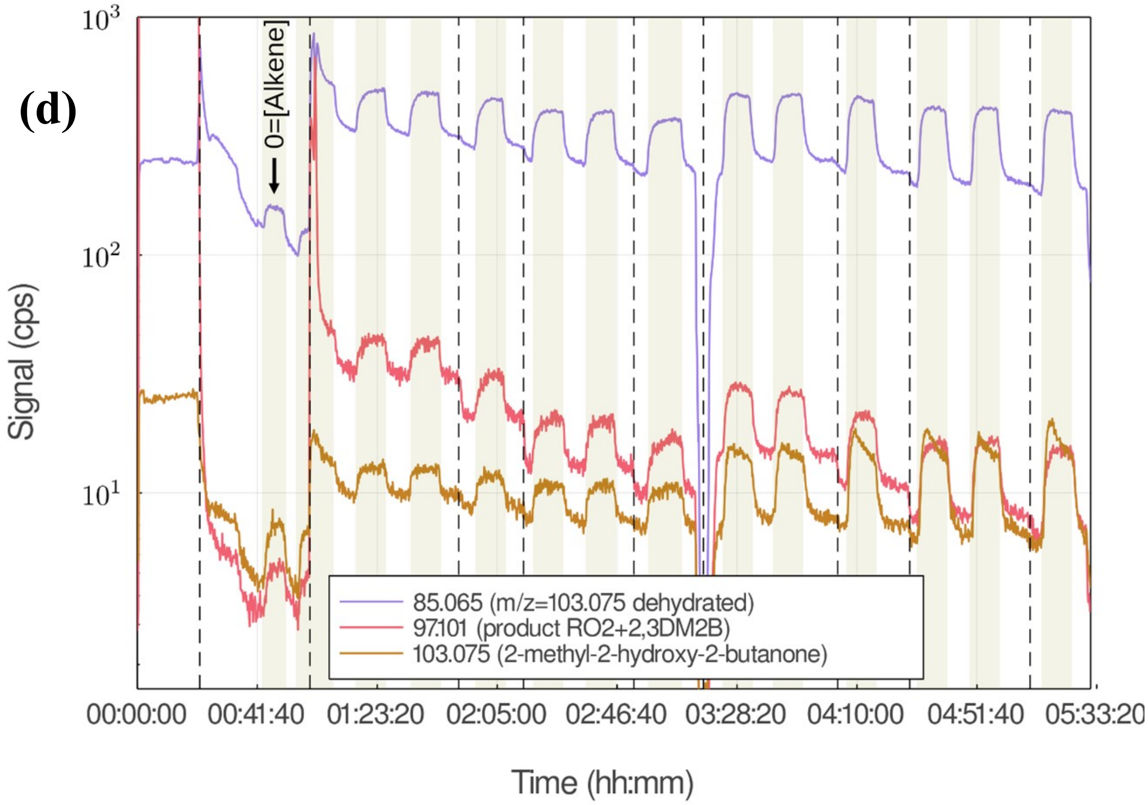

10^3
10^2
10^1
0=[Alkene]
Signal (cps)
00:00:00
00:41:40
01:23:20
02:05:00
02:46:40
03:28:20
04:10:00
04:51:40
05:33:20
Time (hh:mm)
85.065 (m/z=103.075 dehydrated)
97.101 (product RO2+2,3DM2B)
103.075 (2-methyl-2-hydroxy-2-butanone)

(e)

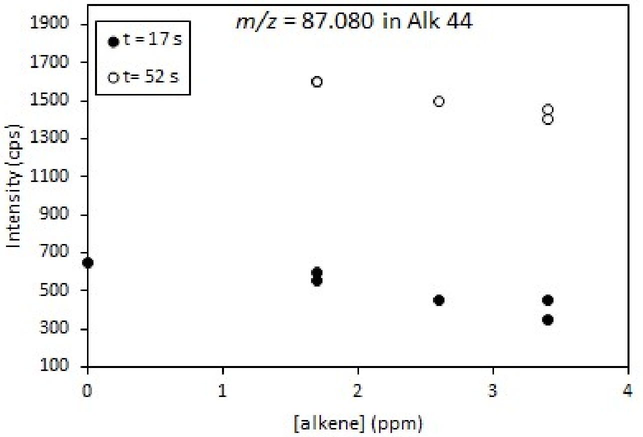
m/z = 87.080 in Alk 44
t = 17 s
t= 52 s
Intensity (cps)
[alkene] (ppm)
1900
1700
1500
1300
1100
900
700
500
300
100
0
1
2
3
4

(f)

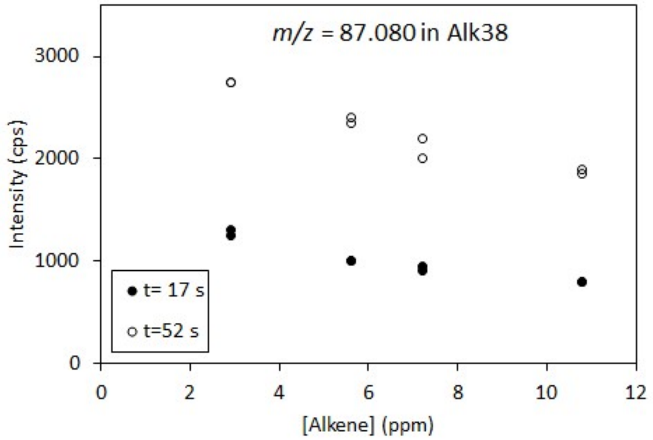
m/z = 87.080 in Alk38
t= 17 s
t=52 s
Intensity (cps)
[Alkene] (ppm)
3000
2000
1000
0
0
2
4
6
8
10
12

(g)

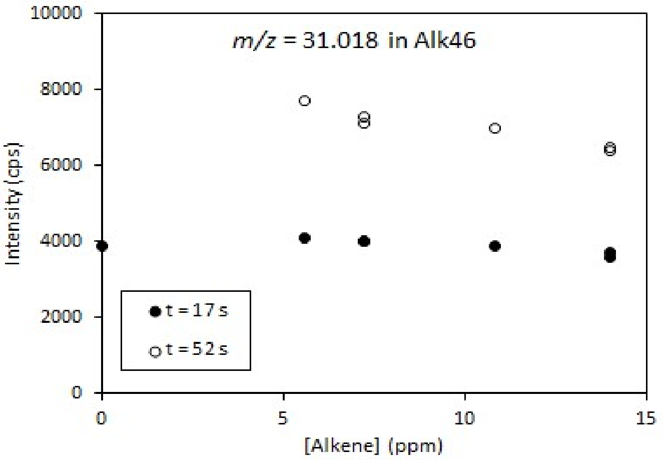
m/z = 31.018 in Alk46
t = 17 s
t = 52 s
Intensity (cps)
[Alkene] (ppm)
10000
8000
6000
4000
2000
0
0
5
10
15

Figure S6: Chromatograms.

Chromatograms obtained in $C_5H_{11}O_2$ + 2,3DM2B experiment (Alk40, sample 4).

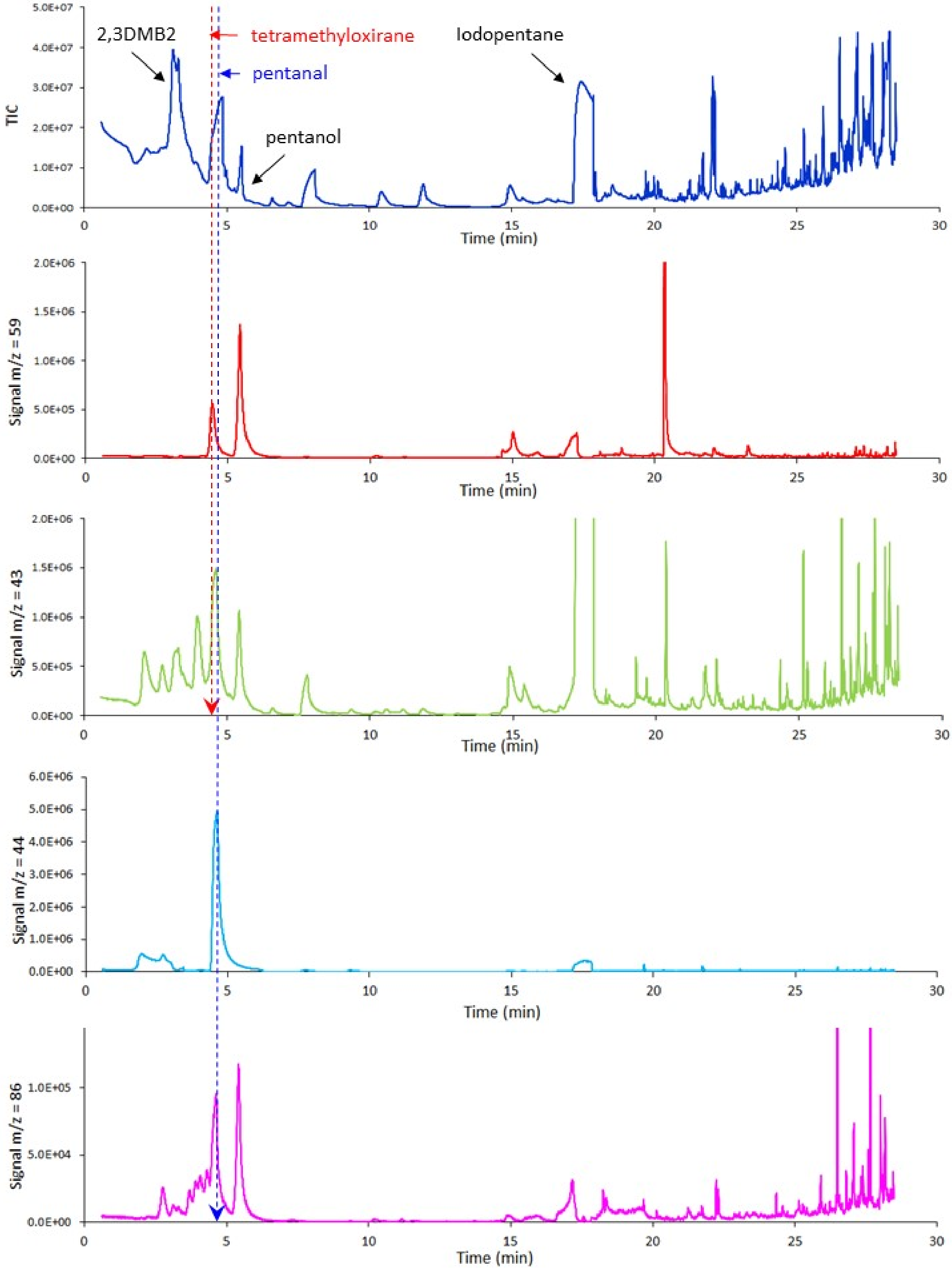

Figure S7: GC/MS analysis: Evolution of the concentration of 1-pentanal and tetramethyloxirane.

A) Pentanal at *m/z* = 44 and 85. B) tetramethyl oxirane at *m/z* = 59. Data are averaged between Alk39 and Alk40 and error bars represent the dispersion between the results. [A] = alkene concentration.

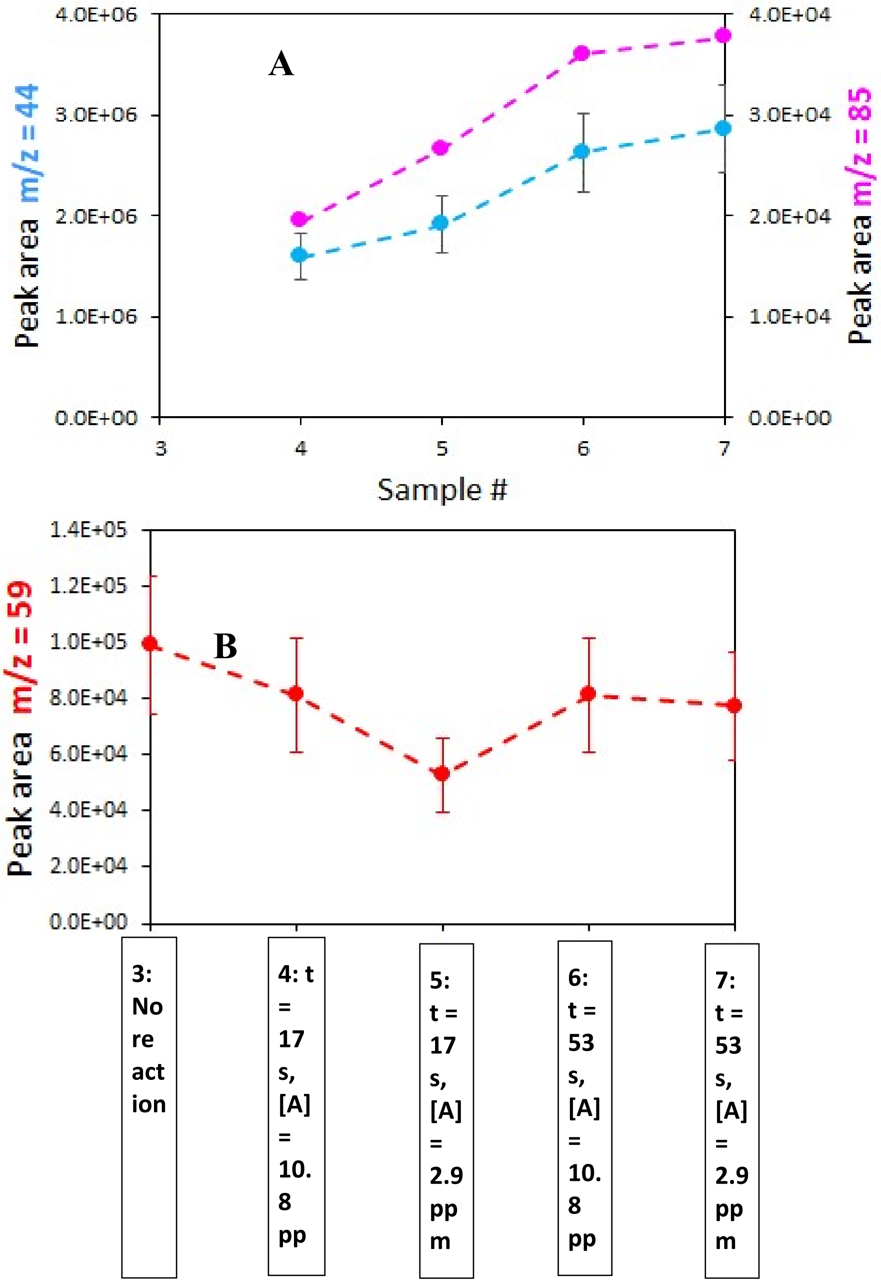

Section S8: **Kinetic simulations**

Kinetic simulations were performed to determine the amount of epoxide expected if RO2+alkene proceeded entirely by the epoxide channel, as suggested in the literature. As in our previous study, ChemSimul V3.90 was used and with the rate coefficients summarized in Table S8.1. The results are presented in Table S8.2, where Δ[alkene] is the amount of alkene consumed and "$HOOQO_2$" the sum of the autoxidation products for $C_5H_{11}O_2$.

Estimation of the detection sensitivity for the epoxides: The detection sensitivity for the epoxides, *So* (cps/ppb) was estimated by considering the background concentration of tetramethyloxirane in Alk38 before reaction. This background signal was *S* ~ 100000 cps (Fig. 3 and above) for a concentration of tetramethyloxirane of 0.2 % x 2.7×10^{14} = 5.4×10^{11} cm^{-3} (22 ppb). Thus, *So* ~ 4630 cps/ppb, which can be rounded up to 5000 cps/ppb and is typical for oxygenated compounds detected by PTR-MS.

Table S8.1: Rate coefficients used in the simulations

Rate coefficients	HO_2	CH_3O_2	$C_5H_{11}O_2$
k_{self} (cm^3 s^{-1})	1.6×10^{-12}	3.5×10^{-13}	4.0×10^{-13}
k_{HO2} (cm^3 s^{-1})	/	5.2×10^{-12}	1×10^{-11}
k_{iso} (s^{-1})	/	/	2.4×10^{-3}
k_{wall} (s^{-1})	3×10^{-3}	3×10^{-3}	3×10^{-3}
k_{alk} (cm^3 s^{-1}) isoprene	1.7×10^{-19}	/	8.0×10^{-18}
k_{alk} (cm^3 s^{-1}) 2,3-dimethyl-2-butene	3.7×10^{-19}	6.7×10^{-18}	1.6×10^{-16}

Table S8.2: Results of the kinetic simulations

All concentrations are in cm^{-3}. The data highlighted in yellow correspond to reaction times close to those studied in the experiments.

$C_5H_{11}O_2$ + 2,3DM2B (Alk38)							
Time (s)	[$C_5H_{11}O_2$]	[1-pentanal]	[HO_2]	[$HOOQO_2$]	[Epoxide]	[Alkene]	Δ[alkene]
0	2.50E+12	0.00E+00	0.00E+00	0.00E+00	0.00E+00	3.00E+14	
6	9.69E+10	1.21E+12	9.58E+09	5.55E+09	1.11E+11	3.00E+14	1.10E+11
12	3.51E+10	1.24E+12	6.59E+09	6.39E+09	1.28E+11	3.00E+14	1.30E+11
18	1.63E+10	1.25E+12	5.58E+09	6.74E+09	1.35E+11	3.00E+14	1.30E+11
24	8.37E+09	1.25E+12	5.04E+09	6.91E+09	1.38E+11	3.00E+14	1.40E+11
30	4.51E+09	1.26E+12	4.66E+09	7.00E+09	1.40E+11	3.00E+14	1.40E+11
36	2.51E+09	1.26E+12	4.35E+09	7.05E+09	1.41E+11	3.00E+14	1.40E+11
42	1.43E+09	1.26E+12	4.08E+09	7.08E+09	1.42E+11	3.00E+14	1.40E+11
48	8.28E+08	1.26E+12	3.84E+09	7.10E+09	1.42E+11	3.00E+14	1.40E+11
54	4.88E+08	1.26E+12	3.61E+09	7.10E+09	1.42E+11	3.00E+14	1.40E+11
60	2.92E+08	1.26E+12	3.40E+09	7.11E+09	1.42E+11	3.00E+14	1.40E+11

$C_5H_{11}O_2$ + isoprene (Alk44)							
Time (s)	[$C_5H_{11}O_2$]	[1-pentanal]	[HO_2]	[$HOOQO_2$]	[Epoxide]	[Alkene]	Δ[Alkene]
0	2.50E+12	0.00E+00	0.00E+00	0.00E+00	0.00E+00	9.00E+13	
6	1.27E+11	1.19E+12	6.89E+09	5.98E+09	2.24E+10	9.00E+13	2.20E+10
12	6.09E+10	1.22E+12	3.81E+09	7.23E+09	2.71E+10	9.00E+13	2.70E+10
18	3.83E+10	1.23E+12	2.75E+09	7.92E+09	2.97E+10	9.00E+13	3.00E+10
24	2.70E+10	1.24E+12	2.22E+09	8.38E+09	3.14E+10	9.00E+13	3.10E+10
30	2.02E+10	1.24E+12	1.90E+09	8.72E+09	3.27E+10	9.00E+13	3.30E+10
36	1.57E+10	1.25E+12	1.69E+09	8.97E+09	3.36E+10	9.00E+13	3.40E+10
42	1.26E+10	1.25E+12	1.54E+09	9.18E+09	3.44E+10	9.00E+13	3.40E+10
48	1.02E+10	1.25E+12	1.43E+09	9.34E+09	3.50E+10	9.00E+13	3.50E+10
54	8.46E+09	1.25E+12	1.34E+09	9.47E+09	3.55E+10	9.00E+13	3.60E+10
60	7.08E+09	1.25E+12	1.27E+09	9.59E+09	3.59E+10	9.00E+13	3.60E+10
CH_3O_2 + 2,3DMB (Alk46)							
Time (s)	[CH_3O_2]	[HCHO]	[HO_2]		[Epoxide]	[Alkene]	Δ[Alkene]
0	5.00E+12	0.00E+00	0.00E+00		0.00E+00	3.50E+14	
6	1.28E+11	2.47E+12	1.27E+10		3.74E+10	3.50E+14	4.00E+10
12	6.17E+10	2.49E+12	6.24E+09		4.38E+10	3.50E+14	4.00E+10
18	3.92E+10	2.50E+12	4.05E+09		4.74E+10	3.50E+14	5.00E+10
24	2.80E+10	2.50E+12	2.95E+09		4.99E+10	3.50E+14	5.00E+10
30	2.12E+10	2.51E+12	2.29E+09		5.17E+10	3.50E+14	5.00E+10
36	1.68E+10	2.51E+12	1.86E+09		5.30E+10	3.50E+14	5.00E+10
42	1.36E+10	2.51E+12	1.55E+09		5.42E+10	3.50E+14	5.00E+10
48	1.13E+10	2.51E+12	1.32E+09		5.51E+10	3.50E+14	6.00E+10
54	9.51E+09	2.51E+12	1.14E+09		5.58E+10	3.50E+14	6.00E+10
60	8.09E+09	2.51E+12	1.00E+09		5.65E+10	3.50E+14	6.00E+10

Figure S9: NMR spectra for the 2,3DM2B and isoprene samples.

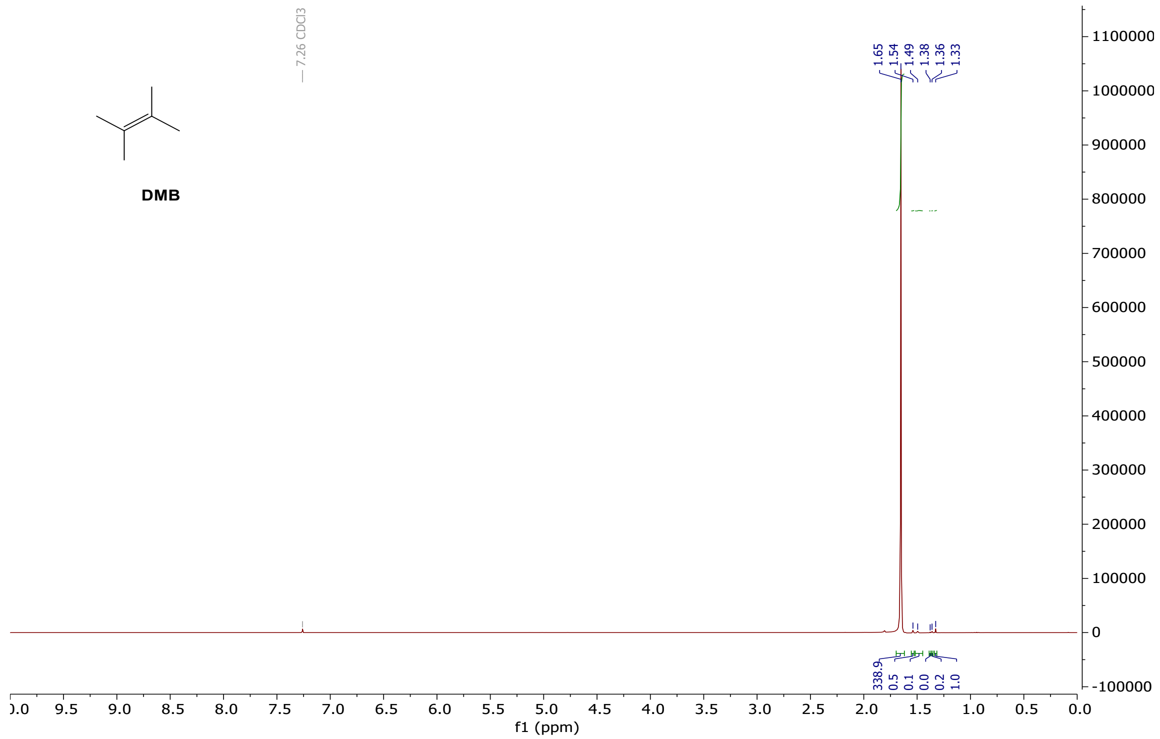

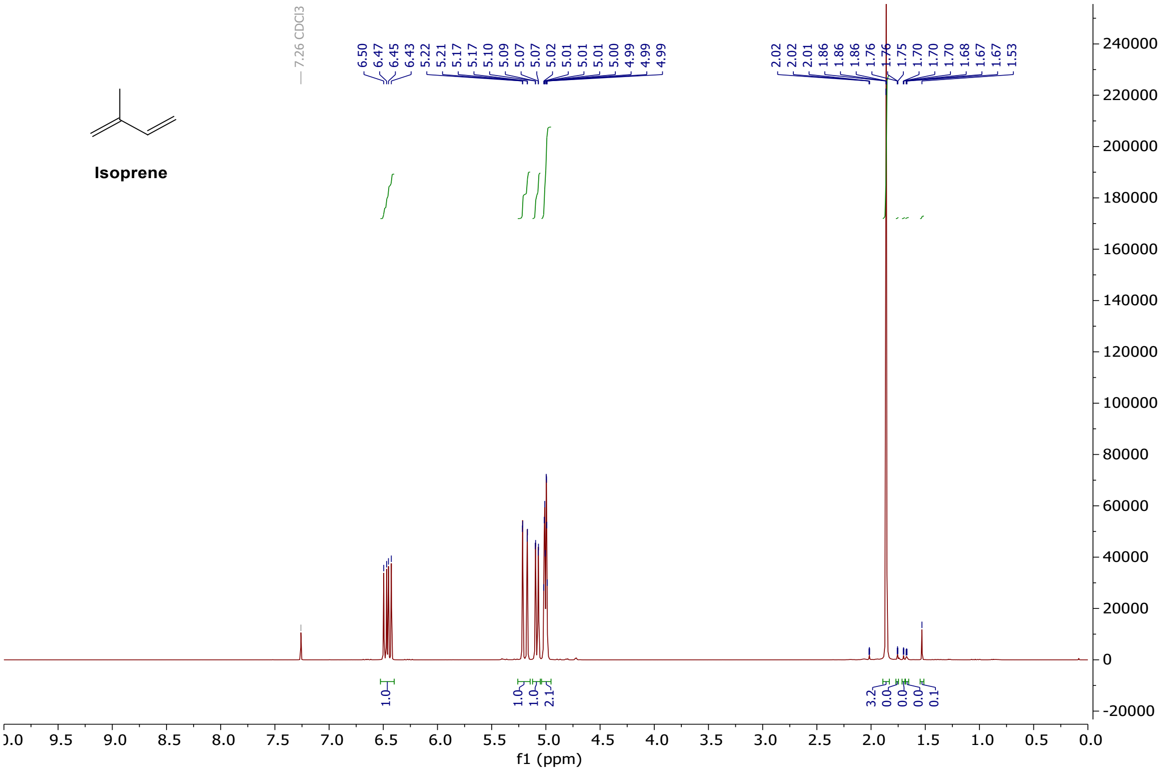

Section S10: Kinetic analyses of the ion signals in the reactions RO_2 + alkene.

A) Kinetic analyses

First-order kinetic analyses were applied to the most intense ion signals attributed to specific RO_2 + Alkene reactions (cf. Tables S2-S4 and discussion in the text). For this, the signal variations in the Light ON / Light OFF cycle *(*ΔS) were measured at the two reaction times explored (t_1 = 17 s, achieved with a flow of 3 sLm and t_2 = 53 s, achieved with a flow of 1 sLm) and for each alkene concentration. The rate coefficient for the formation of the corresponding product, k^{II} ($cm^3\ s^{-1}$), was then obtained by applying the equation:

$$\ln\left(1-\frac{\Delta S1}{\Delta S2}\right) = k^{II} \times [Alkene] \times \left(t_2 - t_1\right) \quad (12).$$

Thus k^{II} was obtained from the slope of ln(1 – ΔS1/ΔS2) as function of [alkene]. In the analyses of the experimental profiles ΔS is measured from the relative baseline. The error bars reported in the plots below represent the uncertainties in measuring ΔS_1 and ΔS_2.

B) Results

Figure S10B1: Evolution of the ion *m/z* = 127.112 in $C_5H_{11}O_2$ + 2,3DM2B experiment (Alk38).

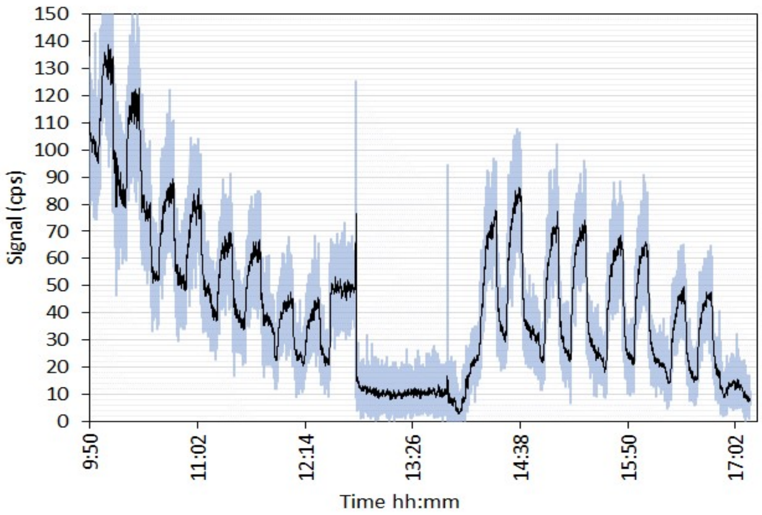

Figure S10B2: Kinetic plot for Fig. S10B1

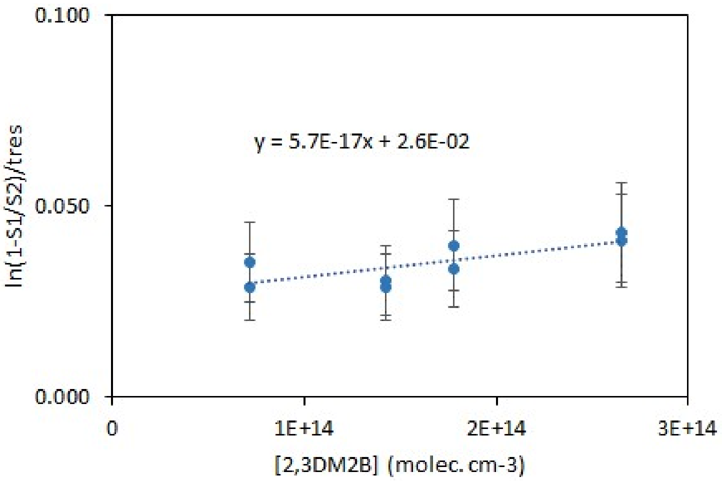

0.100
0.050
0.000
y = 5.7E-17x + 2.6E-02
ln(1-S1/S2)/tres
0
1E+14
2E+14
3E+14
[2,3DM2B] (molec. cm-3)

Figure S10B3: Evolution of the ion *m/z* = 119.034 in $C_5H_{11}O_2$ + isoprene experiment (Alk44).

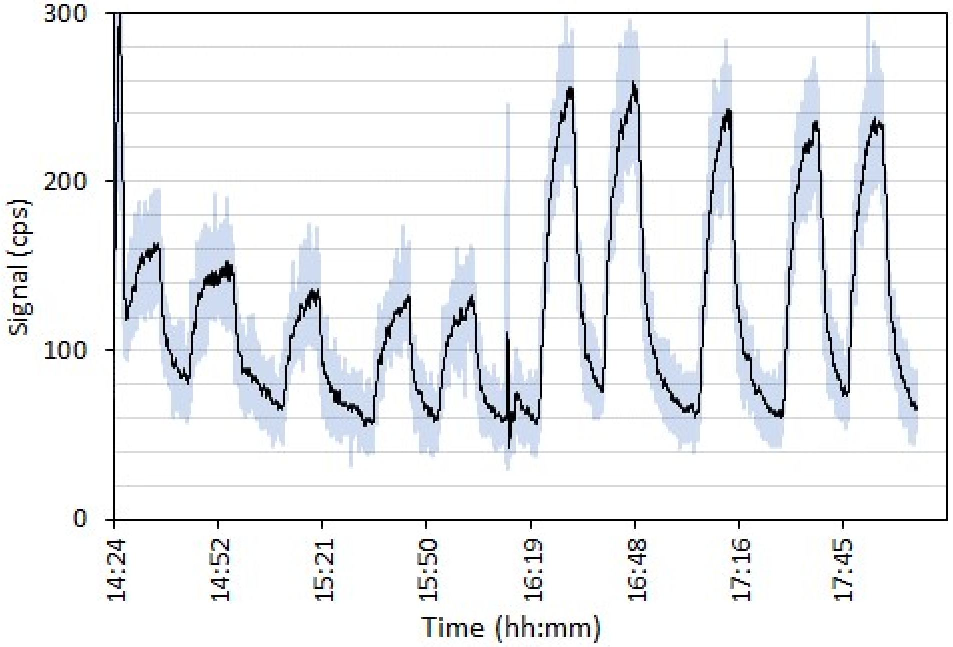

Figure S10B4: Kinetic plot for Fig. S10B3.

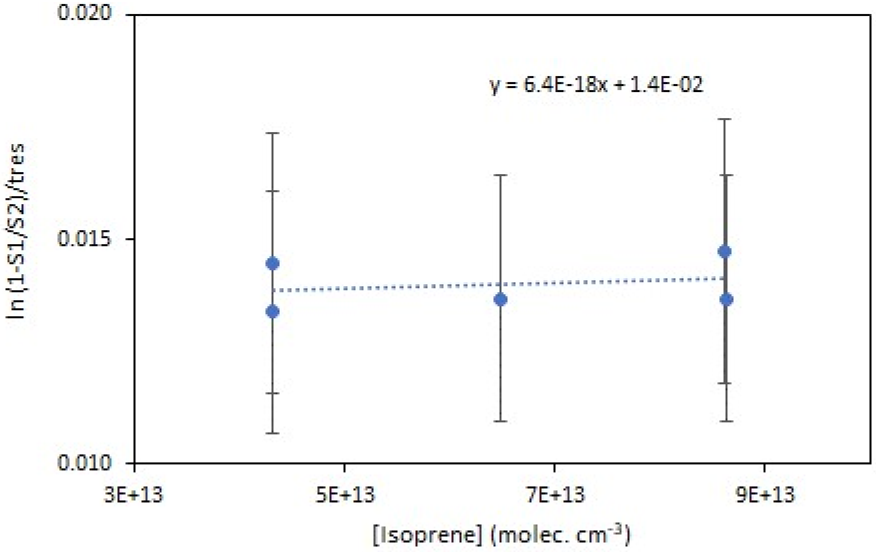

Figure S10B5: Evolution of the ion m/z = 85.065 in CH_3O_2 + 2,3DM2B experiment (Alk46).

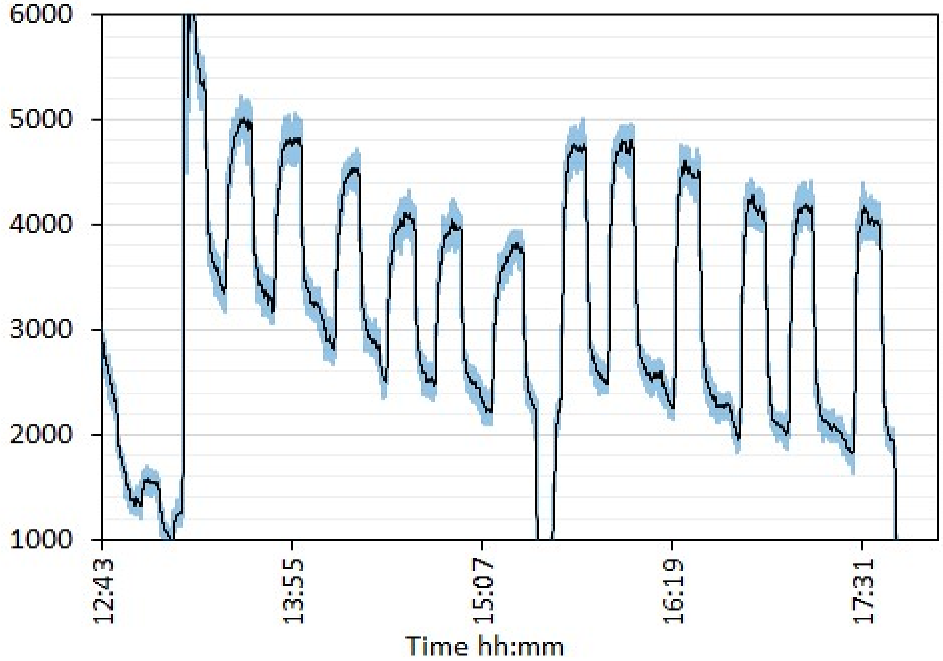

Figure S10B6: Kinetic plot for Fig. S10B5.

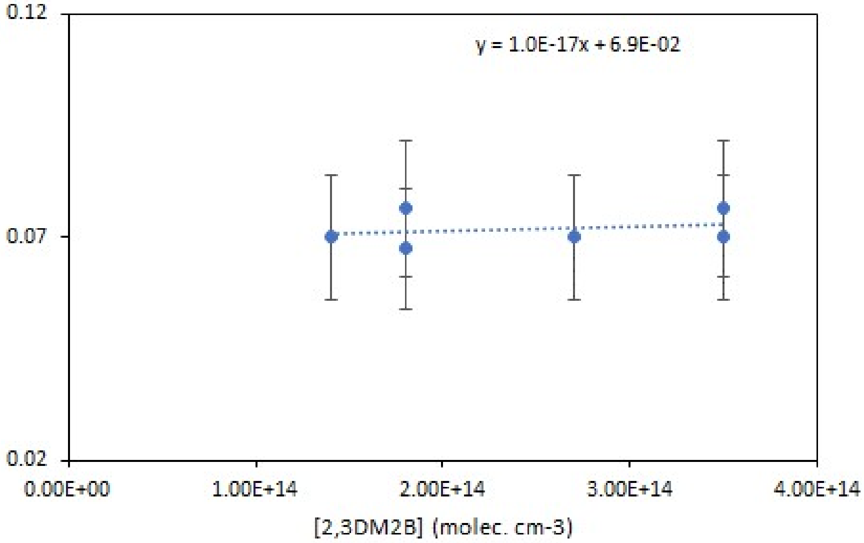

Figure S11: Chromatograms of potential reaction products.

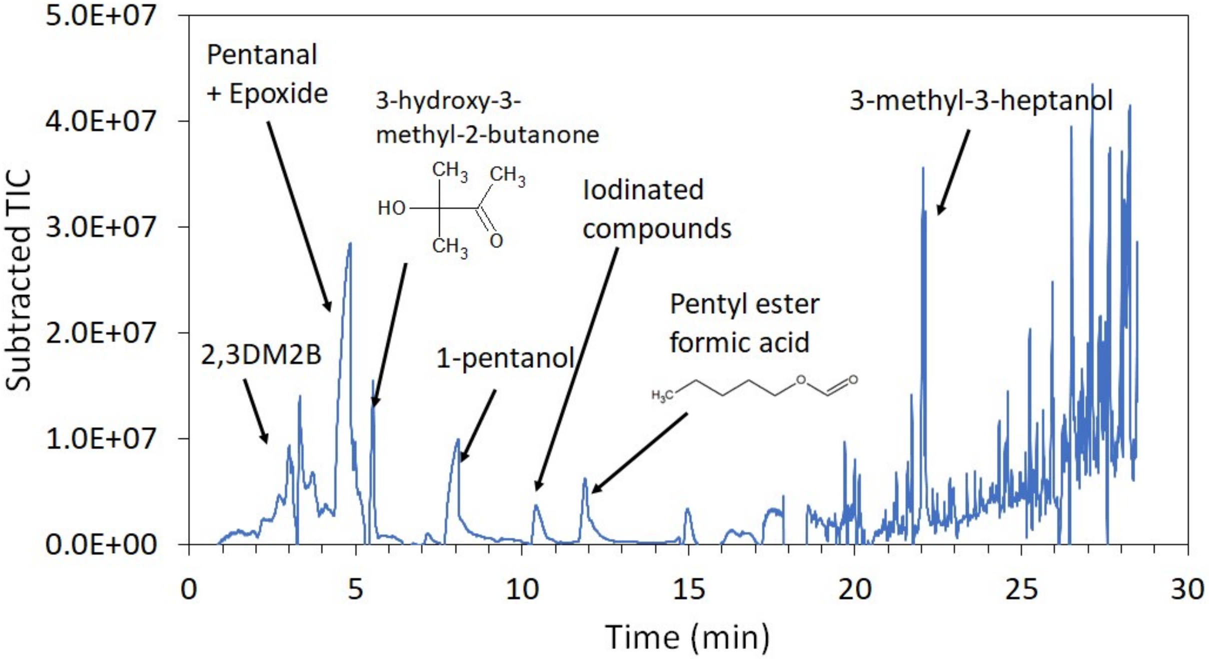

Section S12: Potential effects of RO_2+alkene reactions in chamber experiments (kinetic simulations)

The potential impacts of RO_2+alkene reactions in chamber experiments were examined by using the experiments of ref. 14 (oxidation of isoprene by OH under low NOx conditions) as an example. Simple kinetic simulations were performed using ChemSimul V3.90 and the reactions presented in Table S12.1. The kinetic system was run over 2h-cycles, mimicking the experiments of ref. 14, and kept as simple as possible to examine mostly the effects on the concentration of isoprene-RO_2 ("IsopRO_2"), the total RO_2, ΣRO_2, and OH recycling. To determine a lower limit for the impact of RO_2+isoprene, only one isoprene-RO_2 was considered, Z-δ-RO_2-I, which has the fastest monomolecular sink rate (r_{mono} = 0.1 or 0.4 s^{-1}, see justifications in ref. 14). The initial isoprene concentrations was set to 5 ppb, as in ref. 14 and the rate of photolytic production of OH was adjusted in the simulations to obtain OH concentrations comparable to those reported in ref. 14.

Table S12.1: Reactions and rate coefficients included in the simulations.

Reaction	Rate coefficients	Comment
(1) Precursor $\rightarrow$ OH	8×10^5 cm^{-3} s^{-1}	Photolytic source for OH. Adjusted to fit [OH] to ref. 14
(2) Isoprene +OH $\rightarrow$ IsopOH	1.0×10^{-10} cm^3 s^{-1}	
(3) IsopOH + O_2 $\rightarrow$ IsopRO_2	8.0×10^{-12} cm^3 s^{-1}	
(4) IsopRO_2 $\rightarrow$ DHP-MVK + OH + CO	0.1 to 0.4 s^{-1}	Rates from ref. 14.
(5) IsopRO_2 + IsopRO_2 $\rightarrow$ 2 MVK + 2 HCHO + 2 HO_2	3.0×10^{-12} cm^3 s^{-1}	Source for HO_2
(6) IsopRO_2 +HO_2 $\rightarrow$ ISOPOOH + O_2	1.0×10^{-11} cm^3 s^{-1}	Main sink for HO_2
(7) IsopRO_2 + isoprene $\rightarrow$ C10RO_2	0 to 1.0×10^{-14} cm^3 s^{-1}	Varied to study the impact of RO_2+isoprene
(8) C10RO_2 $\rightarrow$ products	1.0×10^{-2} s^{-1}	Lumped sink rate assumed for the R'O_2 from RO_2+isoprene

Figure S12.2: Results of the simulations: concentrations profiles for isoprene-RO_2 ("IsopRO2"), ΣRO_2, and OH, for two different sink rates for the radical Z-δ-RO_2-I.

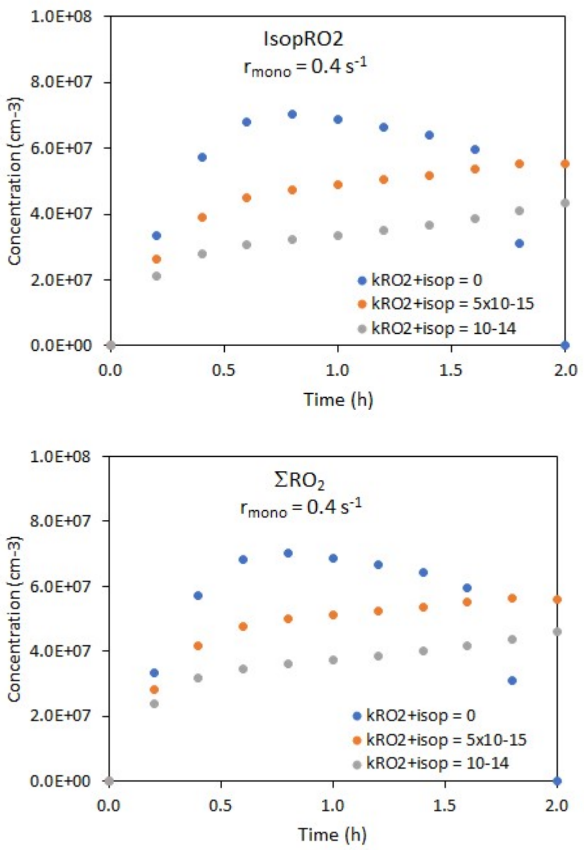

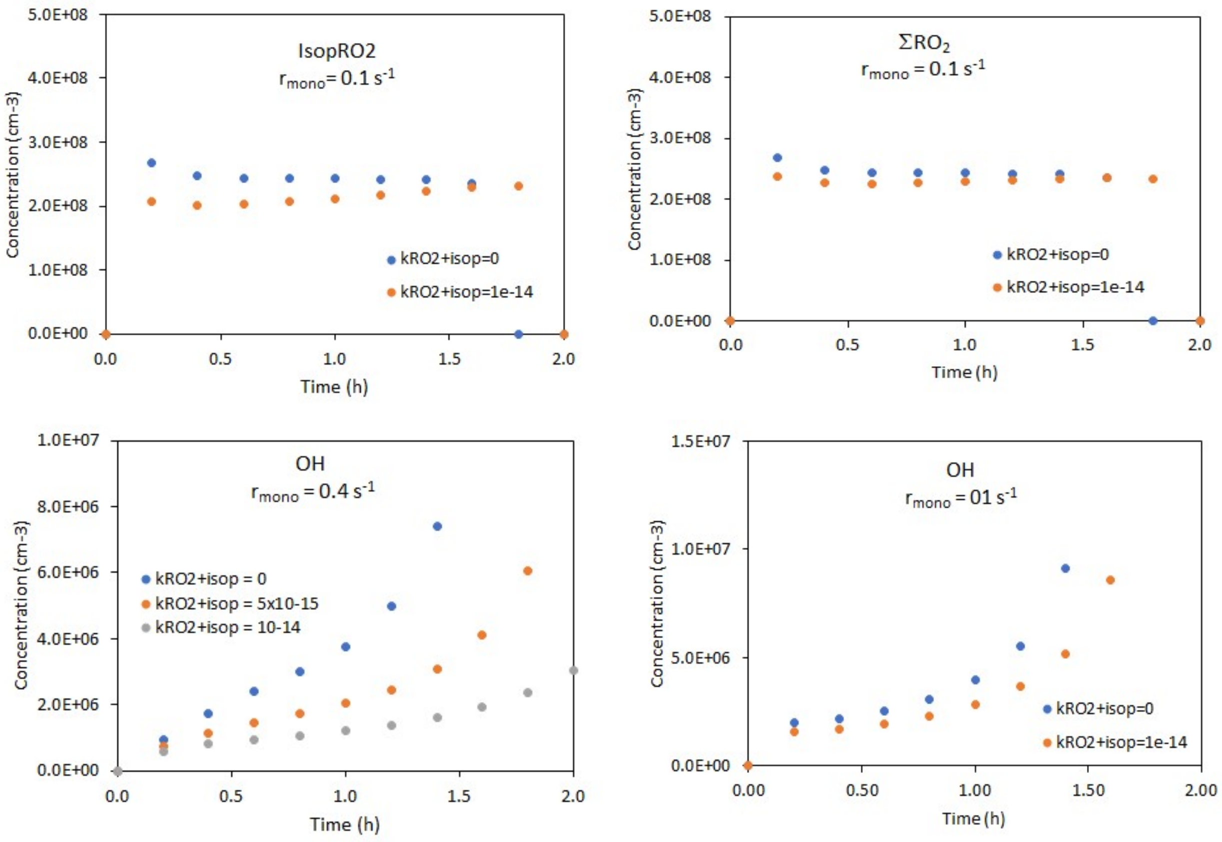
IsopRO2
rmono= 0.1 s-1
kRO2+isop=0
kRO2+isop=1e-14
Concentration (cm-3)
Time (h)
ΣRO2
rmono = 0.1 s-1
kRO2+isop=0
kRO2+isop=1e-14
Concentration (cm-3)
Time (h)
OH
rmono = 0.4 s-1
kRO2+isop = 0
kRO2+isop = 5x10-15
kRO2+isop = 10-14
Concentration (cm-3)
Time (h)
OH
rmono = 01 s-1
kRO2+isop=0
kRO2+isop=1e-14
Concentration (cm-3)
Time (h)
